# A MIG-15/JNK-1 MAP kinase cascade opposes RPM-1 signaling in synapse formation and learning

**DOI:** 10.1371/journal.pgen.1007095

**Published:** 2017-12-11

**Authors:** Oliver Crawley, Andrew C. Giles, Muriel Desbois, Sudhanva Kashyap, Rayna Birnbaum, Brock Grill

**Affiliations:** 1 Department of Neuroscience, The Scripps Research Institute, Scripps Florida, Jupiter, Florida, United States of America; 2 Harriet L. Wilkes Honors College, Florida Atlantic University, Jupiter, FL, United States of America; University of California San Francisco, UNITED STATES

## Abstract

The Pam/Highwire/RPM-1 (PHR) proteins are conserved intracellular signaling hubs that regulate synapse formation and axon termination. The *C*. *elegans* PHR protein, called RPM-1, acts as a ubiquitin ligase to inhibit the DLK-1 and MLK-1 MAP kinase pathways. We have identified several kinases that are likely to form a new MAP kinase pathway that suppresses synapse formation defects, but not axon termination defects, in the mechanosensory neurons of *rpm-1* mutants. This pathway includes: MIG-15 (MAP4K), NSY-1 (MAP3K), JKK-1 (MAP2K) and JNK-1 (MAPK). Transgenic overexpression of kinases in the MIG-15/JNK-1 pathway is sufficient to impair synapse formation in wild-type animals. The MIG-15/JNK-1 pathway functions cell autonomously in the mechanosensory neurons, and these kinases localize to presynaptic terminals providing further evidence of a role in synapse development. Loss of MIG-15/JNK-1 signaling also suppresses defects in habituation to repeated mechanical stimuli in *rpm-1* mutants, a behavioral deficit that is likely to arise from impaired glutamatergic synapse formation. Interestingly, habituation results are consistent with the MIG-15/JNK-1 pathway functioning as a parallel opposing pathway to RPM-1. These findings indicate the MIG-15/JNK-1 pathway can restrict both glutamatergic synapse formation and short-term learning.

## Author summary

We explored the molecular mechanisms that govern synapse formation *in vivo* using the nematode *C*. *elegans*. Our results have identified a conserved MIG-15/JNK-1 MAPK pathway that restricts formation of glutamatergic, neuron-neuron synapses in the mechanosensory neurons, but does not restrict synapse formation in motor neurons. This could have important implications because synapses made by the mechanosensory neurons are reminiscent of synapses in the mammalian central nervous system, and relatively little is known about the signals that specifically influence central synapse formation *in vivo*. Interestingly, our results are consistent with the MIG-15/JNK-1 pathway opposing RPM-1, a signaling hub and ubiquitin ligase that inhibits different JNK and p38 signaling pathways. This suggests RPM-1 might inhibit specific MAPK pathways, such as the DLK-1 pathway, rather than simply acting as a general inhibitor of JNK and p38 signaling in neurons. Our results are particularly interesting given emerging links between JNK MAPK signaling and neurodegenerative diseases, such as Alzheimer’s disease.

## Introduction

Information is relayed throughout the nervous system via chemical synapses, and the process of synapse formation is essential for construction of neural circuitry [1, 2]. In the central nervous system, most synaptic connections are glutamatergic and often referred to as central synapses.

Different neurons in the nematode *C*. *elegans* have proven extremely valuable in identifying conserved regulators of synapse formation, including motor neurons, HSN neurons, and mechanosensory neurons [3, 4]. Of these, only the mechanosensory neurons form glutamatergic, neuron-neuron synapses that are reminiscent of central synapses. Genetic screens using mechanosensory neurons have revealed several molecules that regulate glutamatergic synapse formation including the intracellular signaling hub Regulator of Presynaptic Morphology 1 (RPM-1) [5], the F-box protein MEC-15 [6], the transcription factor SAM-10 [7], the focal adhesion protein ZYX-1/Zyxin [8], and the microRNA LIN-4 [9].

*C*. *elegans* has two PLM mechanosensory neurons, which sense posterior gentle touch [10]. The PLM neurons form electrical synapses and glutamatergic chemical synapses with postsynaptic interneurons [10–12]. Initial touch sensation is thought to rely upon both electrical synapses and glutamatergic transmission. Glutamatergic transmission is also required for more complex touch-response behaviors, such as habituation to repeated tap and arousal from a sleep-like state called lethargus [13, 14].

RPM-1 is the *C*. *elegans* ortholog of mouse Phr1 and Drosophila Highwire, which are collectively referred to as Pam/Highwire/RPM-1 (PHR) proteins. RPM-1 is a relatively broad regulator of synapse formation and axon termination affecting these processes in mechanosensory neurons and motor neurons [5, 15–17]. Studies in flies and mice have shown these are conserved RPM-1 functions [18–22]. Previous work showed RPM-1 functions in the mechanosensory neurons during development to regulate habituation to repeated tap stimulus, a behavioral deficit that likely results from abnormal glutamatergic synapse formation in *rpm-1* mutants [23].

PHR proteins are enormous intracellular signaling hubs that regulate numerous downstream pathways [15]. Proteomic screens identified several RPM-1 binding proteins that mediate RPM-1 function during development including: the RCC1-like protein GLO-4 [24], the microtubule binding protein RAE-1 [25, 26], the PP2C phosphatase PPM-2 [27], and the Nesprin ANC-1 [28]. Suppressor genetics revealed that RPM-1 inhibits p38 and JNK MAPK signaling [29–31]. Studies from flies and mammals have shown this is a conserved PHR protein function [32–34]. RPM-1 inhibits MAPK signaling by ubiquitinating MAP3Ks, such as DLK-1 and MLK-1, thereby targeting them for degradation by the proteasome [29, 35, 36]. At present, it remains unclear whether RPM-1 is a general inhibitor of MAP3Ks during neuronal development, or a potentially selective regulator of certain p38 and JNK pathways.

JNK MAP kinase pathways play a conserved role in synapse formation in the developing nervous system [37]. Studies in worms [29, 30], flies [32, 38] and vertebrates [39, 40] have shown JNK signaling needs to be restricted for proper synapse formation. The role of JNK signaling in synapse formation has been primarily explored using the neuromuscular junction (NMJ) in different organisms. Much less is known about how JNK signaling impacts the formation of neuron-neuron, central synapses *in vivo*. It also remains unclear if JNK isoforms have differential roles in mediating synapse formation in different types of neurons. The importance of addressing these gaps in our knowledge is highlighted by emerging links between altered JNK signaling and synaptic dysfunction in Alzheimer’s disease [41–44].

JNK activation is regulated by upstream kinase cascades that include MAP2Ks, MAP3Ks, and in some cases MAP4Ks. *In vitro* biochemistry and genetic studies on fly embryogenesis have shown Misshapen (Msn), called MIG-15 in worms and NIK (HGK/MAP4K4) in mammals, can function as a MAP4K that activates JNK signaling [45–47]. While MIG-15 and Msn regulate axon guidance in the nervous system, they do so through the cytoskeletal regulators ERM-1 and Bifocal [48, 49]. To date, functional links in the nervous system between JNK signaling and MIG-15 or Msn have remained stubbornly elusive.

Using suppressor genetics, we identified several kinases that are likely to be part of a new JNK pathway that regulates glutamatergic, neuron-neuron synapse formation in the mechanosensory neurons of *C*. *elegans*. This JNK pathway is composed of MIG-15 (MAP4K), NSY-1 (MAP3K), JKK-1 (MAP2K), and JNK-1. Loss of function mutations in kinases of the MIG-15/JNK-1 pathway specifically suppress defects in synapse formation in the mechanosensory neurons of *rpm-1* mutants. Consistent with suppression results, transgenic overexpression of kinases in the MIG-15/JNK-1 pathway in wild-type animals results in impaired synapse formation. Further biochemical support for the MIG-15/JNK-1 pathway comes from our observation that NSY-1 can bind both MIG-15 and JKK-1 when expressed in HEK 293 cells.

Based on prior work, the most likely signaling model that might explain our results is RPM-1 acting as a ubiquitin ligase to inhibit an upstream kinase in the MIG-15/JNK-1 pathway, similar to RPM-1 effects on the DLK-1 and MLK-1 pathways. However, our behavioral analysis of habituation indicated the MIG-15/JNK-1 pathway is more likely to function as a parallel opposing pathway to RPM-1. These results provide the first potential link in the nervous system between the MIG-15 MAP4K and a JNK isoform, and indicate MIG-15/JNK-1 activity can affect synapse formation and short-term learning.

## Results

### Synapse formation defects in *rpm-1* mutants, but not axon termination defects, are suppressed by *jkk-1* and *jnk-1*

Previous studies showed that loss-of-function mutations in kinases of the DLK-1/PMK-3 p38 MAPK pathway and the MLK-1/KGB-1 JNK pathway suppress synapse formation defects in *rpm-1* mutants [29, 30]. A different pathway consisting of JNK-1 and an MKK7 ortholog JKK-1 functions in the nervous system to regulate locomotion, and synaptic vesicle trafficking in GABAergic motor neurons [50, 51]. *jkk-1* and *jnk-1* also suppress synaptic position defects in cholinergic NMJs of *arl-8* mutants [52]. These observations prompted us to test whether *jkk-1* and *jnk-1* could suppress defects in synapse formation and axon termination caused by *rpm-1* loss of function (lf) in the mechanosensory neurons.

**Fig 1:**
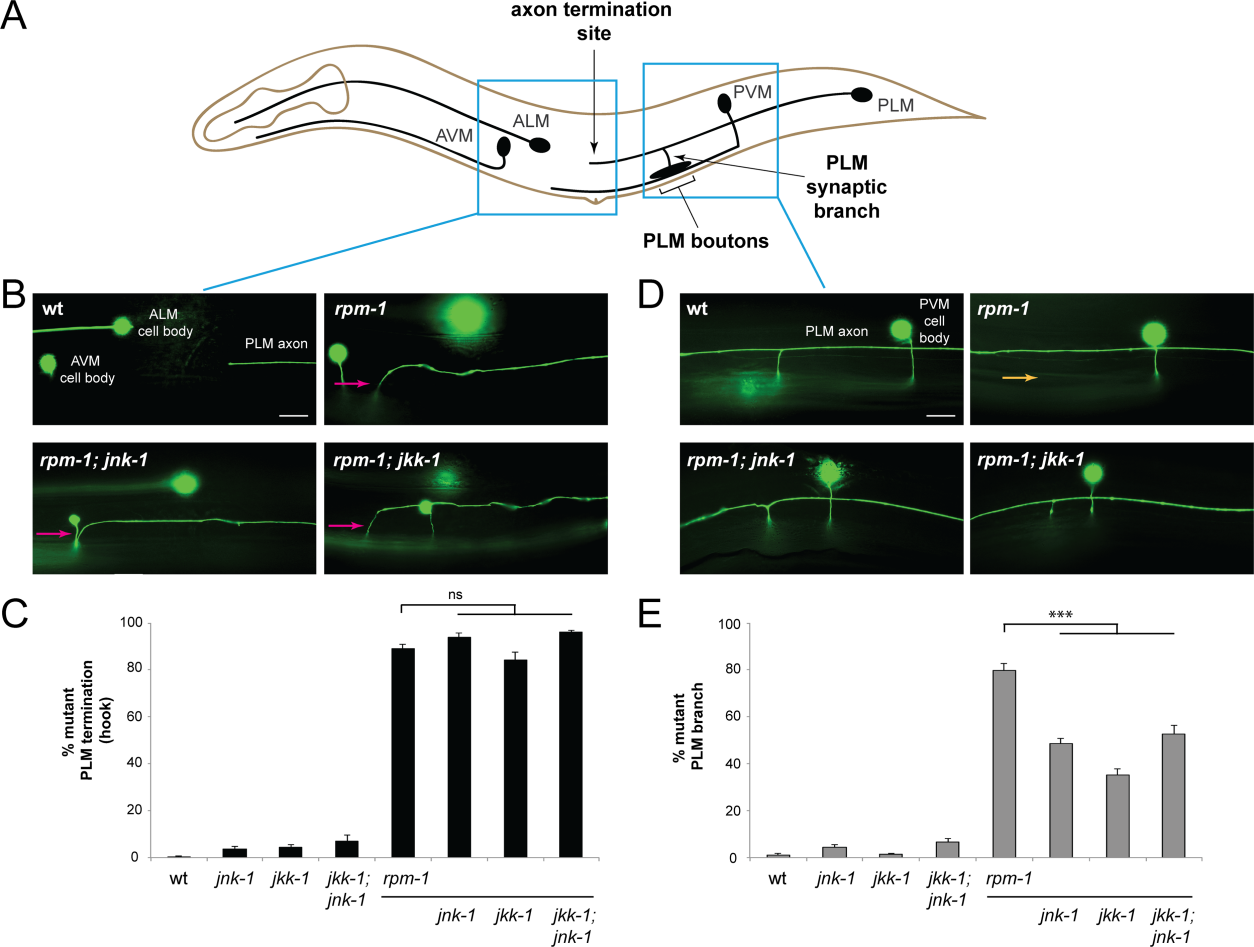
Loss of *jnk-1* and *jkk-1* suppress synapse formation defects, but not axon termination defects, in *rpm-1* mutants. (**A**) Schematic showing *C*. *elegans* mechanosensory neurons. Left blue box highlights PLM axon termination site. Right blue box highlights collateral synaptic branch and presynaptic boutons. (**B**) Transgene *muIs32*, which expresses GFP in mechanosensory neurons, was used to visualize axon termination in a wild-type animal. Axon termination defects (hook defect) in an *rpm-1* single mutant, and *rpm-1; jnk-1* and *rpm-1; jkk-1* double mutants (magenta arrows). (**C**) Quantitation of axon termination defects (hook defect) for indicated genotypes. (**D**) PLM synaptic branch in wildtype. Synaptic branch is absent in *rpm-1* mutant (orange arrow), a defect that results from failed synapse formation. In *rpm-1; jnk-1* and *rpm-1; jkk-1* double mutants synaptic branch is present indicating *jnk-1* and *jkk-1* suppress this defect. Note presynaptic boutons are below synaptic branch, and out of focal plane. (**E**) Quantitation of synaptic branch defects for indicated genotypes. Averages are shown from 6–10 counts (25–30 neurons/count) of young adult animals for each genotype. Error bars represent standard error of mean, and significance was determined using unpaired Student’s *t* test with Bonferroni correction. *** p< 0.001, ns = not significant. Scale bars 10 μm.

*C*. *elegans* has two PLM mechanosensory neurons, one on each side of its body. Each PLM neuron extends a single axon that terminates growth prior to the cell body of the ALM mechanosensory neuron (Fig 1A and 1B). Glutamatergic chemical synapses are formed *en passant* with interneurons by a collateral branch that extends from the primary axon (Fig 1A and 1D). The transgene *muIs32*, which expresses GFP in the mechanosensory neurons, can be used to assess PLM neurons for axon termination, as well as formation of the synaptic branch and synaptic boutons [24]. Anatomical separation of axon termination and chemical synapse formation makes the PLM neurons an ideal system to assess these two developmental processes within the same neuron.

Consistent with prior studies [5, 53], we observed that *rpm-1* mutants display two phenotypes in the PLM neurons. The first is failed axon termination, which results in the PLM axon extending beyond the ALM cell body and hooking towards the ventral side of the animal, which we refer to as a hook defect (Fig 1B). The second phenotype is retraction of the synaptic branch, which results from impaired synapse formation (Fig 1D) [5]. Quantitation of axon termination defects, and synaptic branch defects in *rpm-1* mutants are shown in Fig 1C and 1E, respectively.

To test if *jkk-1* and *jnk-1* affect axon termination and synapse formation of PLM neurons, we generated double mutants with previously described null alleles for *jkk-1 (km2)* and *jnk-1 (gk7)* [50, 54]. *rpm-1; jkk-1* and *rpm-1; jnk-1* double mutants did not show changes in the frequency of axon termination defects (Fig 1B and 1C). In contrast, synaptic branch defects were significantly suppressed in both double mutants (Fig 1D and 1E).

Given that both *jkk-1* and *jnk-1* suppress synaptic branch defects, but not axon termination defects, we tested whether they function in the same genetic pathway. To do so, we generated *rpm-1*; *jkk-1*; *jnk-1* triple mutants. We observed no further suppression of synaptic branch defects in triple mutants (Fig 1E), and again saw no suppression of axon termination defects (Fig 1C). These results are consistent with *jkk-1* and *jnk-1* functioning in the same genetic pathway to specifically suppress synapse formation defects in the PLM neurons of *rpm-1* mutants.

### MIG-15 and NSY-1 function in the JKK-1/JNK-1 pathway to regulate synapse formation

**Fig 2:**
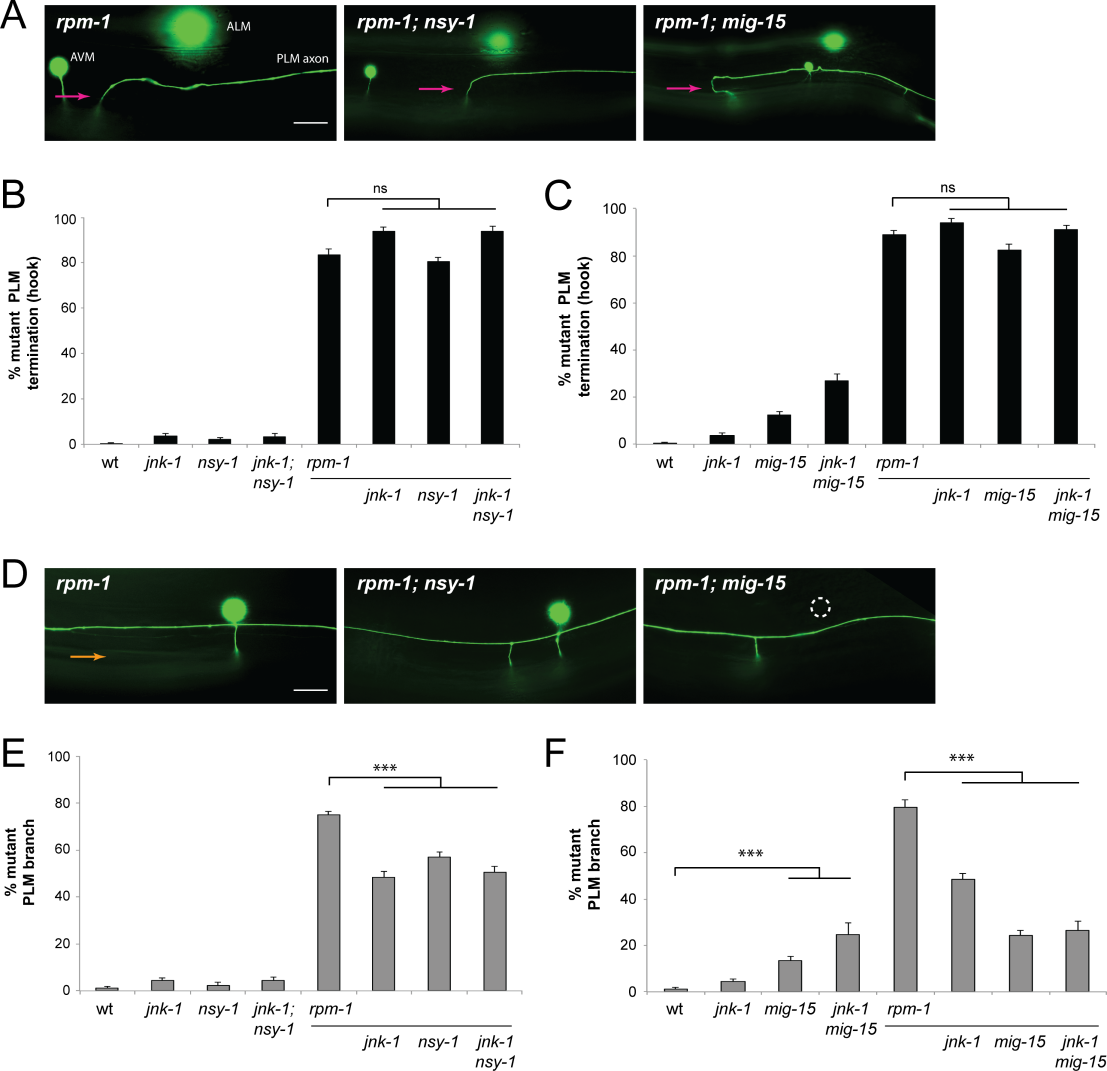
*nsy-*1 and *mig-15* suppress synapse formation defects in *rpm-1* mutants. (**A**) Shown are PLM axon termination defects (magenta arrows) in *rpm-1* single mutants, as well as *rpm-1; nsy-1* and *rpm-1; mig-15* double mutants. Note *mig-15* results in small body size. (**B-C**) Quantitation of axon termination defects for indicated genotypes. (**D**) Shown is a synaptic branch defect in an *rpm-1* mutant (orange arrow), which is suppressed in *rpm-1; nsy-1* and *rpm-1; mig-15* double mutants. Dashed white circle notes migration defect in which the PVM cell body is absent in *mig-15*. (**E-F**) Quantitation of synaptic branch defects for indicated genotypes. Averages are shown from 4–10 counts (25–30 neurons/count) of young adult animals for each genotype. Error bars represent standard error of mean, and significance was determined using unpaired Student’s *t* test with Bonferroni correction. *** p< 0.001, ns = not significant. Scale bars 10 μm.

Next, we explored MAP3Ks and MAP4Ks that might exist in the JKK-1/JNK-1 pathway. We began by testing the MAP3K NSY-1 because it regulates olfactory neuron fate during development [55], and its mammalian ortholog ASK activates JNK signaling *in vitro* [56]. To analyze genetic interactions between *rpm-1* and *nsy-1*, we used a previously described *nsy-1* allele, *ok593*, that deletes the kinase domain and is likely to be a null. Similar to outcomes with *jkk-1* and *jnk-1*, we observed suppression of synaptic branch defects in *rpm-1; nsy-1* double mutants, but did not see changes in axon termination defects (Fig 2A and 2D). Quantitation showed that suppression of synaptic branch defects in *rpm-1; nsy-1* double mutants was significant compared to *rpm-1* single mutants (Fig 2E). No significant decrease in axon termination defects occurred in *rpm-1; nsy-1* double mutants (Fig 2B). Importantly, no increased suppression occurred in *rpm-1*; *jnk-1; nsy-1* triple mutants compared to either double mutant (Fig 2E). These results are consistent with *nsy-1* functioning in the same pathway as *jnk-1* to suppress synapse formation defects.

Previous studies on MAP kinases regulating neuronal development in *C*. *elegans* have often identified three component pathways (MAP3K, MAP2K and MAPK) [29–31]. At present, potential MAP4Ks that regulate JNK signaling in neuronal development remain unknown. MIG-15 is orthologous to mammalian NIK and fly Msn, which can act as MAP4Ks in cultured cells and during embryogenesis [46, 47]. In the developing *C*. *elegans* nervous system, MIG-15 regulates cell migration and axon guidance, but functional genetic links to JNK signaling in neurons remain absent [57, 58]. Therefore, we tested if loss of MIG-15 function specifically suppresses synapse formation defects in *rpm-1* mutants similar to *nsy-1*, *jkk-1* and *jnk-1*. We used a strong loss of function allele, *mu342*, that is a point mutation in the MIG-15 kinase domain and likely to disrupt kinase activity [58]. Axon termination defects were not suppressed in *rpm-1; mig-15* double mutants (Fig 2A and 2C). However, synaptic branch defects were significantly suppressed in *rpm-1; mig-15* double mutants (Fig 2D and 2F). It should be noted that in some *mig-15* mutants, the PVM cell body did not migrate normally, which is consistent with previously described cell migration defects (Fig 2D). Similar to results with *nsy-1*, further suppression of branch defects was not observed in *rpm-1*; *jnk-1*; *mig-15* triple mutants compared to *rpm-1; mig-15* double mutants. This result is consistent with *mig-15* functioning in the same genetic pathway as *jnk-1*.

The logic of how MAPK modules operate suggests the most likely order for this putative kinase pathway from most upstream to downstream kinase would be: MIG-15/NSY-1/JKK-1/JNK-1. However, our genetic results do not rule out the alternative possibility that these kinases are part of multiple MAPK pathways that function in parallel to suppress synapse formation defects, but not axon termination defects, caused by *rpm-1* (lf).

In addition, we observed a low but significant level of synaptic branch defects in *mig-15* single mutants (Fig 2F). This observation, and suppression of synaptic branch defects caused by *rpm-1* (lf) suggest that a balance of MIG-15 activity is required for proper synapse formation in the PLM neurons.

### Loss of *mig-15* or *jkk-1* results in improved bouton morphology and accumulation of presynaptic components in *rpm-1* mutants

Previous studies suggested impaired synapse formation in *rpm-1* mutants leads to retraction of the PLM synaptic branch [5]. Therefore, the absence of the synaptic branch served as a proxy for assessing synapse formation in different double mutants of *rpm-1* and kinases of interest. However, there are two possible cellular explanations for why synaptic branch defects are suppressed. The first is that synapse formation is improved. Alternatively, MIG-15, NSY-1, JKK-1 and JNK-1 kinases might be required for synaptic branch retraction.

**Fig 3:**
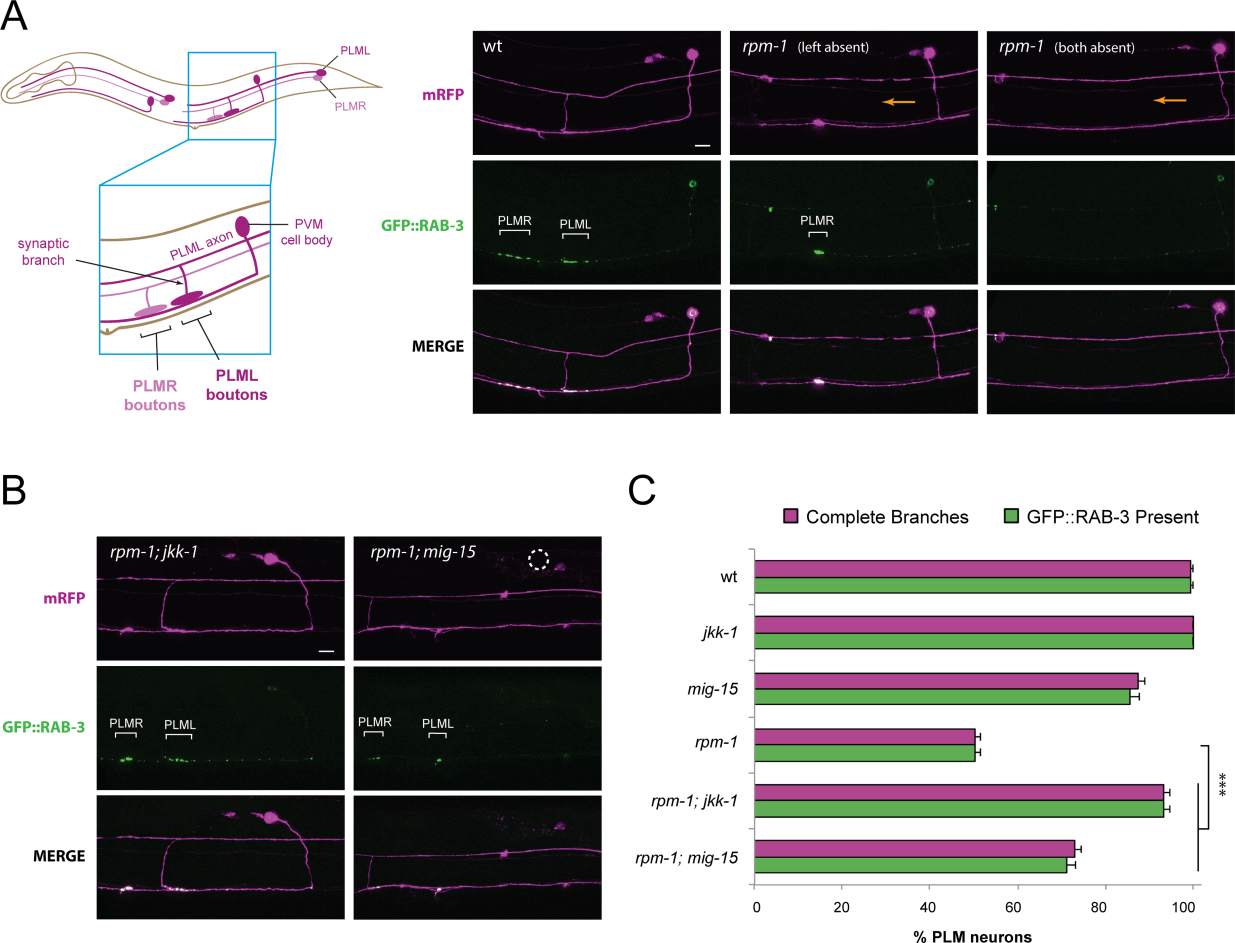
Synaptic vesicle marker RAB-3 is present at presynaptic terminals of *rpm-1; jkk-1* and *rpm-1; mig-15* double mutants. (**A**) Schematic shows mechanosensory neurons on left (shaded dark) and right side (shaded lighter). Blue box zooms in on PLML and PLMR collateral branches which form presynaptic boutons with the ventral nerve cord. Note PVM neuron is not the post-synaptic target of PLM neurons. Shown is a confocal projection of the PLM axon and collateral branch labeled with mRFP (magenta) and GFP::RAB-3 (green) labeling presynaptic boutons (white brackets) in wildtype. Shown are two examples of *rpm-1* mutants in which one or both PLM synaptic branches (orange arrow) and presynaptic boutons (absence of white brackets) are missing. Note PLMR synaptic branch is out of focal plane in middle projection. (**B**) Confocal projection of *rpm-1; jkk-1* and *rpm-1; mig-15* double mutants showing synaptic branch and GFP::RAB-3 labeled presynaptic terminals (white brackets). Dashed white circle annotates absence of PVM cell body in *mig-15* mutant. (**C**) Quantitation of frequency of complete synaptic branches and presence of GFP::RAB-3 at presynaptic boutons for indicated genotypes. Shown are averages from 6–8 counts (25–30 neurons/count) of young adult animals for each genotype. Error bars represent standard error of mean, and significance was determined using unpaired Student’s *t* test with Bonferroni correction. ** p<0.01, *** p< 0.001. Scale bars 10 μm.

To differentiate between these two possibilities, we simultaneously labeled PLM neurons with two transgenes: 1) *jsIs1114* which expresses the synaptic vesicle marker GFP::RAB-3, and 2) *jsIs973* which expresses mRFP and allows the morphology of the axon, synaptic branch and synaptic boutons to be visualized. RAB-3 is a small G-protein that associates with synaptic vesicles (SV), and has been used to assess synaptogenesis in PLM neurons [8]. Consistent with prior work, we observed GFP::RAB-3 at the presynaptic boutons of wild-type animals (Fig 3A). In confocal projections, it is possible to see synaptic boutons from both the left and right PLM neurons (PLML and PLMR) as they reside in the same z-plane in the ventral nerve cord. However, the morphology of only a single PLM axon can be visualized (Fig 3A). We observed two phenotypic groups of *rpm-1* mutants. The first, and most common, lacked one of the two PLM synaptic branches. The example shown in Fig 3A (*rpm-1* left absent) is missing the left PLM synaptic branch and accompanying GFP::RAB-3 accumulations, but GFP::RAB-3 from PLMR is still present. Less frequently, we observed *rpm-1* mutants in which both PLM neurons lack a synaptic branch and show no presynaptic GFP::RAB-3 accumulation (Fig 3A, *rpm-1* both absent). Quantitation showed accumulation of RAB-3 always corresponded with the presence of a synaptic branch in wild-type animals (Fig 3C). The frequency of complete synaptic branches, defined as branches with presynaptic boutons and GFP::RAB-3 accumulation, were reduced in *rpm-1* mutants (Fig 3C). In *rpm-1; mig-15* and *rpm-1; jkk-1* double mutants, we observed an increase in the number of complete synaptic branches and corresponding accumulation of GFP::RAB-3 at presynaptic terminals (Fig 3B and 3C). Thus, *mig-15* and *jkk-1* suppression of *rpm-1* results in improved synapse formation.

### *jnk-1*, *jkk-1* and *nsy-1* enhances defects in synapse formation caused by colchicine

Loss of function in NSY-1, JKK-1 and JNK-1 had minimal effects on PLM synapse formation, as assessed by the presence of the synaptic branch and presynaptic boutons (Figs 1E and 2E). Nonetheless, it was possible more subtle changes might exist in morphology or number of presynaptic terminals when these kinases are perturbed.

**Fig 4:**
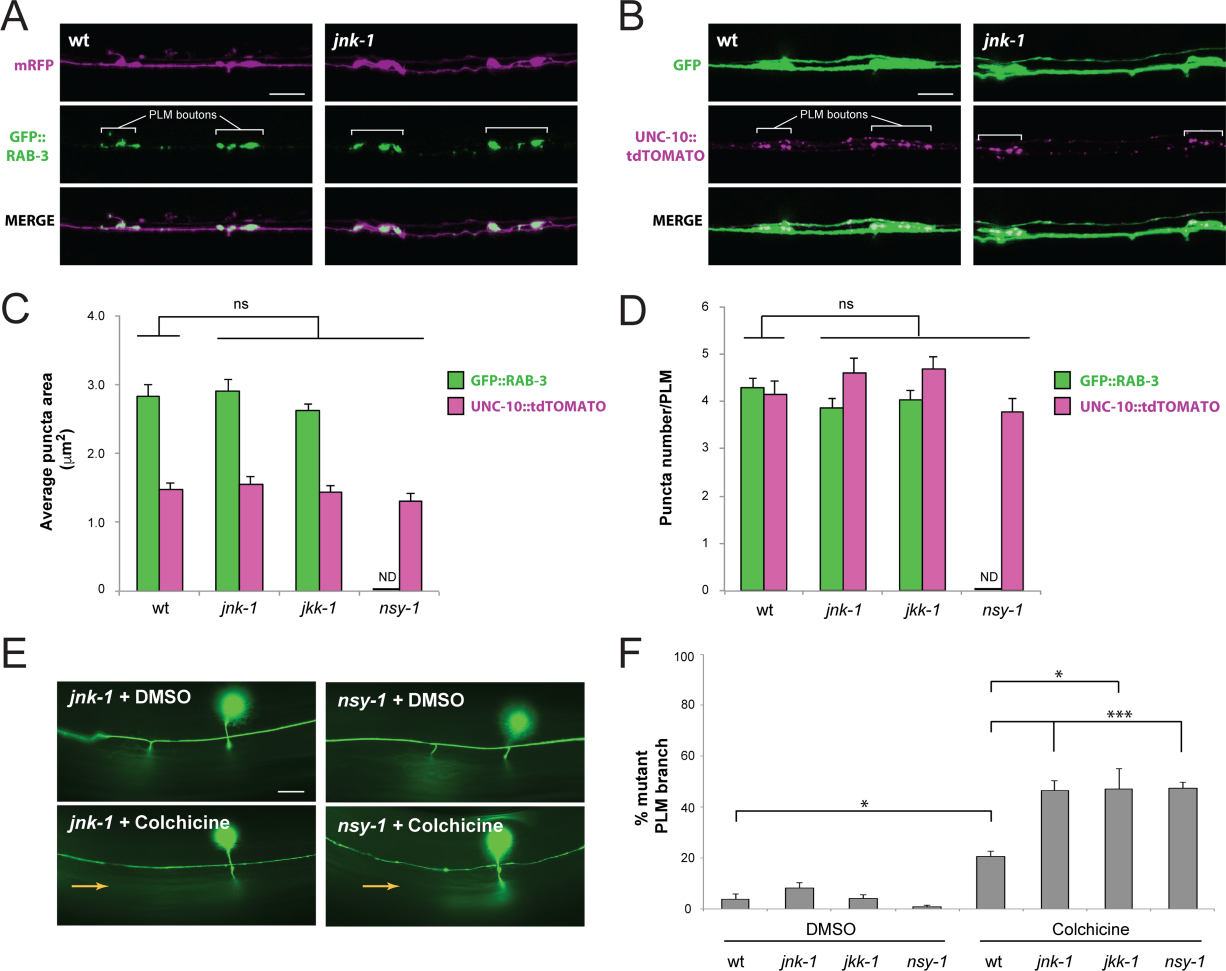
*jnk-1*, *jkk-1* and *nsy-1* loss of function enhances synapse formation defects caused by colchicine. (**A**) Confocal projections of PLM presynaptic boutons labeled with GFP::RAB-3 (green) and mRFP (magenta) for indicated genotypes. (**B**) Confocal projections of PLM presynaptic boutons labeled with UNC-10::tdTOMATO (magenta) and GFP (green). (**C**) Quantitation of GFP::RAB-3 and UNC-10::tdTOMATO puncta area. (**D**) Quantitation of puncta number for GFP::RAB-3 and UNC-10::tdTOMATO. For **C** and **D**, averages are shown from 37–50 animals per genotype. (**E**) Synaptic branch (orange arrow) is absent in *jnk-1* and *nsy-1* mutant treated with colchicine. (**F**) Quantitation of synaptic branch defects for indicated genotypes treated with colchicine or DMSO. Averages are shown from 7–9 counts (25–35 neurons/count). Significance was determined using unpaired Student’s *t* test with Bonferroni correction. * p<0.05, *** p< 0.001 ND = not determined. Scale bars 10 μm.

To test this, we utilized transgenic worms that express either the synaptic vesicle marker GFP::RAB-3, or the active zone marker UNC-10::tdTOMATO in the mechanosensory neurons (Fig 4A and 4B). Transgenes with an appropriate cell fill marker (GFP or mRFP) were evaluated simultaneously to anatomically identify PLM presynaptic terminals. Both presynaptic RAB-3 and UNC-10 puncta size and number showed no significant differences when wild-type animals were compared to *jnk-1*, *jkk-1* or *nsy-1* mutants (Fig 4C and 4D). Data for *nsy-1* and GFP::RAB-3 is not shown because recombinants between *nsy-1* and the RAB-3 transgene could not be obtained.

To further evaluate whether these kinases might affect synapse formation outside of *rpm-1* suppression, we turned to the microtubule destabilizing drug colchicine. It was previously shown that treating wild-type worms with colchicine causes synapse formation defects in PLM neurons [59]. This provided us with a pharmacological manipulation to further examine the role of the MIG-15/JNK-1 pathway in synapse formation. While no effect was observed with the vehicle DMSO, we found that low frequency synaptic branch defects caused by colchicine were enhanced in *jnk-1*, *jkk-1* or *nsy-1* mutants (Fig 4E and 4F). These observations indicate altering microtubule stability can also unveil a role for NSY-1, JKK-1 and JNK-1 in glutamatergic synapse formation.

### The MIG-15/JNK-1 pathway functions cell autonomously in mechanosensory neurons to regulate synapse formation

**Fig 5:**
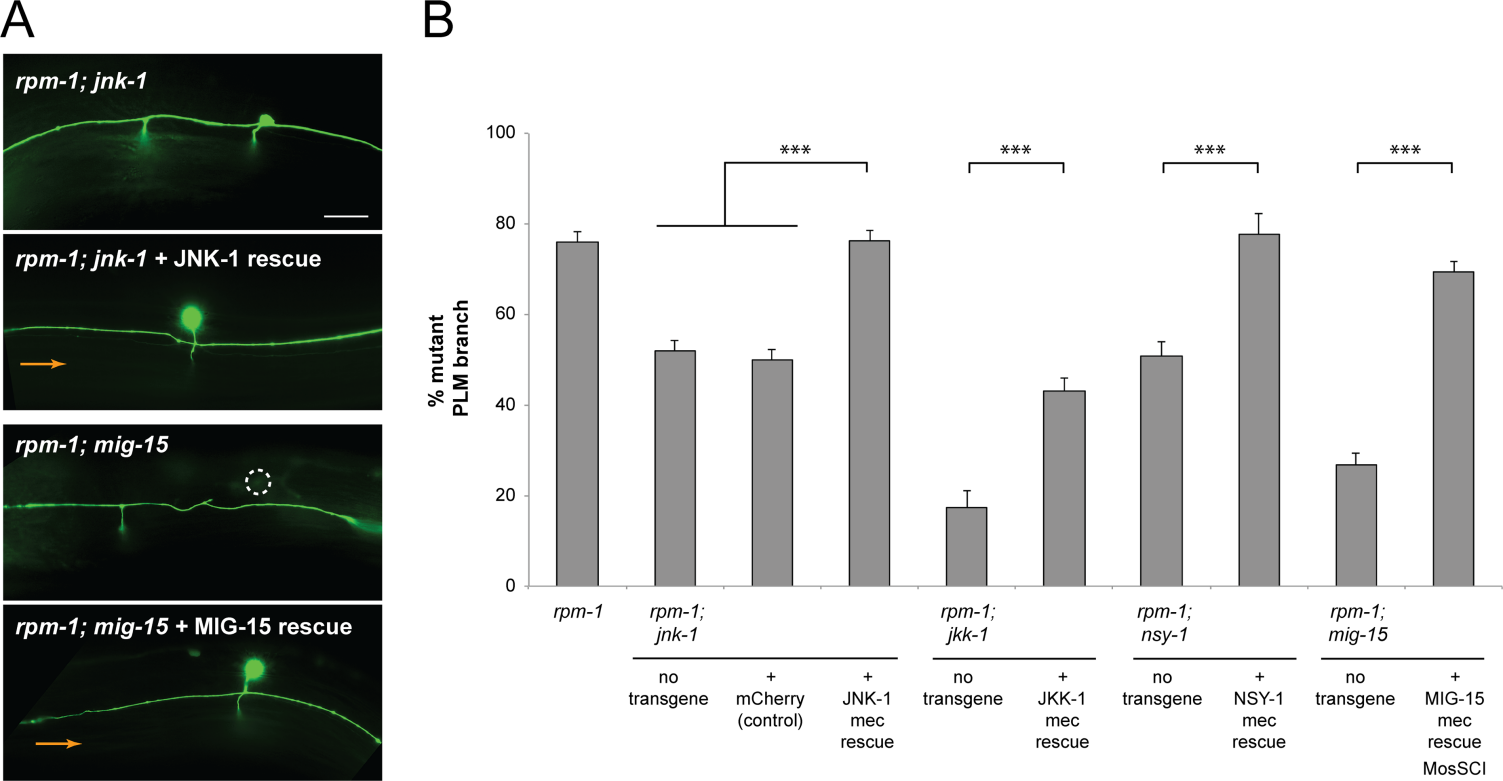
MIG-15/JNK-1 pathway functions cell autonomously in PLM neurons to regulate synapse formation. (**A**) Shown are images of PLM axon and synaptic branch for indicated genotypes. In *rpm-1; jnk-1* and *rpm-1; mig-15* double mutants, transgenic expression of JNK-1 (JNK-1 rescue) and MIG-15 (MIG-15 rescue) in mechanosensory neurons rescues suppression of synaptic branch defects (orange arrows). (**B**) Quantitation of synaptic branch defects for indicated genotypes. Averages are shown for data from 4–6 independent transgenic lines for each genotype. Error bars represent standard error of mean, and significance was determined using unpaired Student’s *t* test with Bonferroni correction. *** p< 0.001. Scale bar 10 μm.

Our genetic analysis showed *mig-15*, *nsy-1*, *jkk-1* and *jnk-1* are likely to function in a linear genetic pathway to suppress synapse formation defects in *rpm-1* mutants. Next, we wanted to determine if these kinases regulate synapse formation by functioning cell autonomously within PLM neurons, or non-cell autonomously in surrounding tissue. We addressed this with transgenic rescue experiments using double mutants of *rpm-1* and different kinases. Transgenic expression of kinases was driven by the *mec-7* promoter, which is expressed in mechanosensory neurons including the PLM neurons, and is not expressed in the postsynaptic interneurons or tissues that surround the PLM synapse, such as muscles or hypodermis. NSY-1, JKK-1 and JNK-1 were expressed using transgenic extrachromasomal arrays. MIG-15 was expressed using a MosSCI single-copy integrated transgene because *mig-15* mutants have small body and brood size, which made deriving extrachromosomal arrays extremely difficult. Expression of each kinase in PLM neurons gave significant, robust rescue of suppression in kinase double mutants with *rpm-1* (Fig 5A and 5B). For example, transgenic expression of JNK-1 (but not the negative control protein mCherry) in *rpm-1; jnk-1* double mutants significantly rescued suppression of synaptic branch defects (Fig 5A and 5B). Our findings support the conclusion that MIG-15, NSY-1, JKK-1 and JNK-1 function cell autonomously in PLM mechanosensory neurons, and are consistent with these kinases functioning in a linear cascade to regulate synapse formation.

### Overexpression of MIG-15, NSY-1 and JKK-1 is sufficient to impair synapse formation

**Fig 6:**
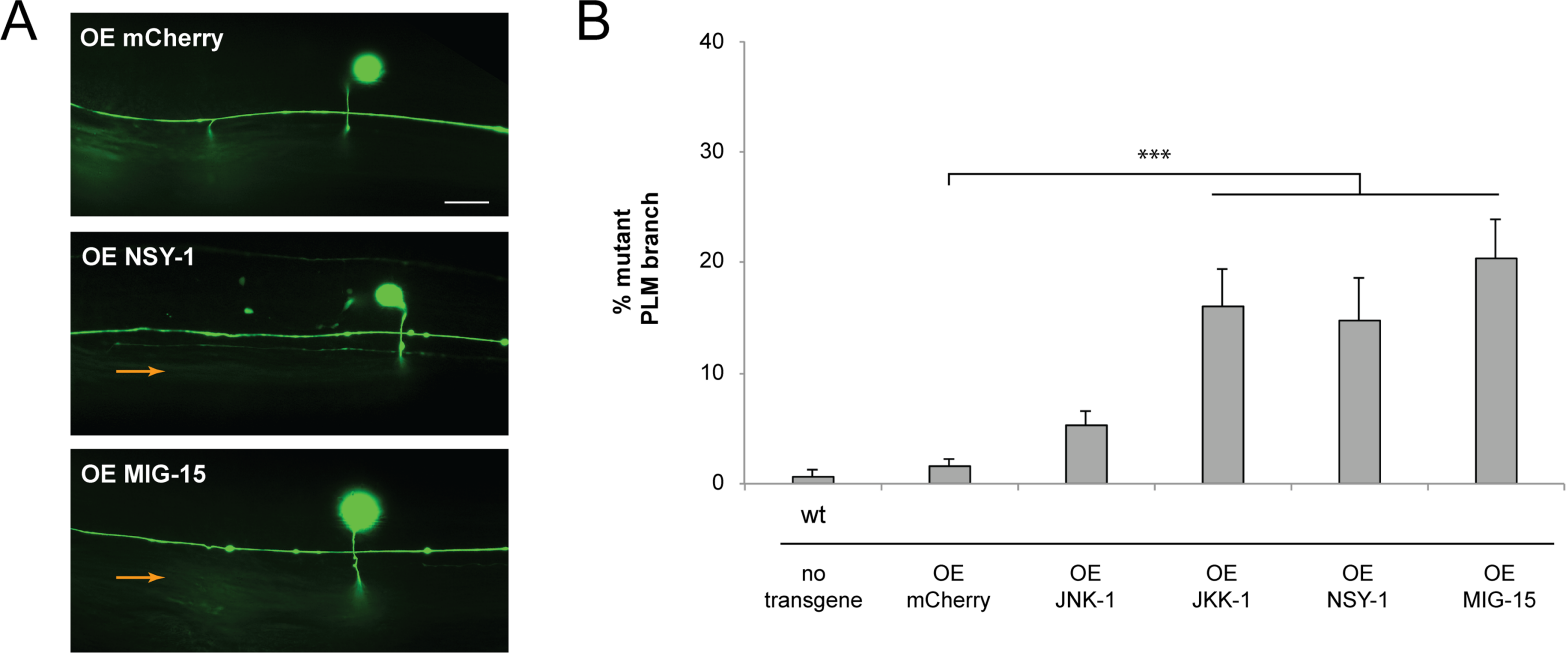
Overexpression of MIG-15, NSY-1, or JKK-1 is sufficient to impair synapse formation. (**A**) Shown is PLM axon and synaptic branch of transgenic animal overexpressing mCherry (OE mCherry). Synapse formation defects (orange arrows) occur when NSY-1 or MIG-15 are overexpressed. (**B**) Quantitation of PLM synaptic branch defects for indicated genotypes. Averages are shown for data from 5 or more independent transgenic lines for each genotype. Error bars represent standard error of mean, and significance was determined using unpaired Student’s *t* test with Bonferroni correction. *** p< 0.001. Scale bar 10 μm.

Our observation that loss of MIG-15, NSY-1, JKK-1 or JNK-1 improves synapse formation defects in *rpm-1* mutants suggests these kinases might inhibit synapse formation. To test this hypothesis, we transgenically overexpressed individual kinases in wild-type animals using the pan-neuronal *rgef-1* promoter, which has been used previously for transgenic overexpression experiments with PLM neurons [30, 53]. PLM synaptic branches were not altered in wild-type animals carrying transgenic extrachromosomal arrays that overexpress mCherry, which was used as a negative control (Fig 6A and 6B). In contrast, transgenic overexpression of MIG-15, NSY-1 or JKK-1 in wild-type worms resulted in synaptic branch defects (Fig 6A and 6B). Transgenic overexpression of JNK-1 did not lead to defects (Fig 6B). This result with JNK-1, which is likely at the bottom of a possible MIG-15/JNK-1 pathway, is not surprising given the prior finding that kinases such as the p38 MAPK PMK-3 and the JNK isoform KGB-1 generate relatively low frequency defects when transgenically overexpressed [30]. Nonetheless, our results show that overexpressing MIG-15, NSY-1 or JKK-1 is sufficient to impair synapse formation. This indicates that these kinases need to be opposed functionally or restricted for proper PLM synapse formation to occur. Furthermore, synapse formation defects caused by overexpression of MIG-15, NSY-1 and JKK-1 occurred at similar levels, which provides further evidence these kinases could function in the same MAPK pathway.

### NSY-1 MAP3K binds to both MIG-15 MAP4K and JKK-1 MAP2K

Our genetic experiments suggested that MIG-15, NSY-1, JKK-1 and JNK-1 potentially function in a novel MAPK pathway that regulates synapse formation in PLM neurons. We wanted to further evaluate the biochemistry that underpins these findings.

**Fig 7:**
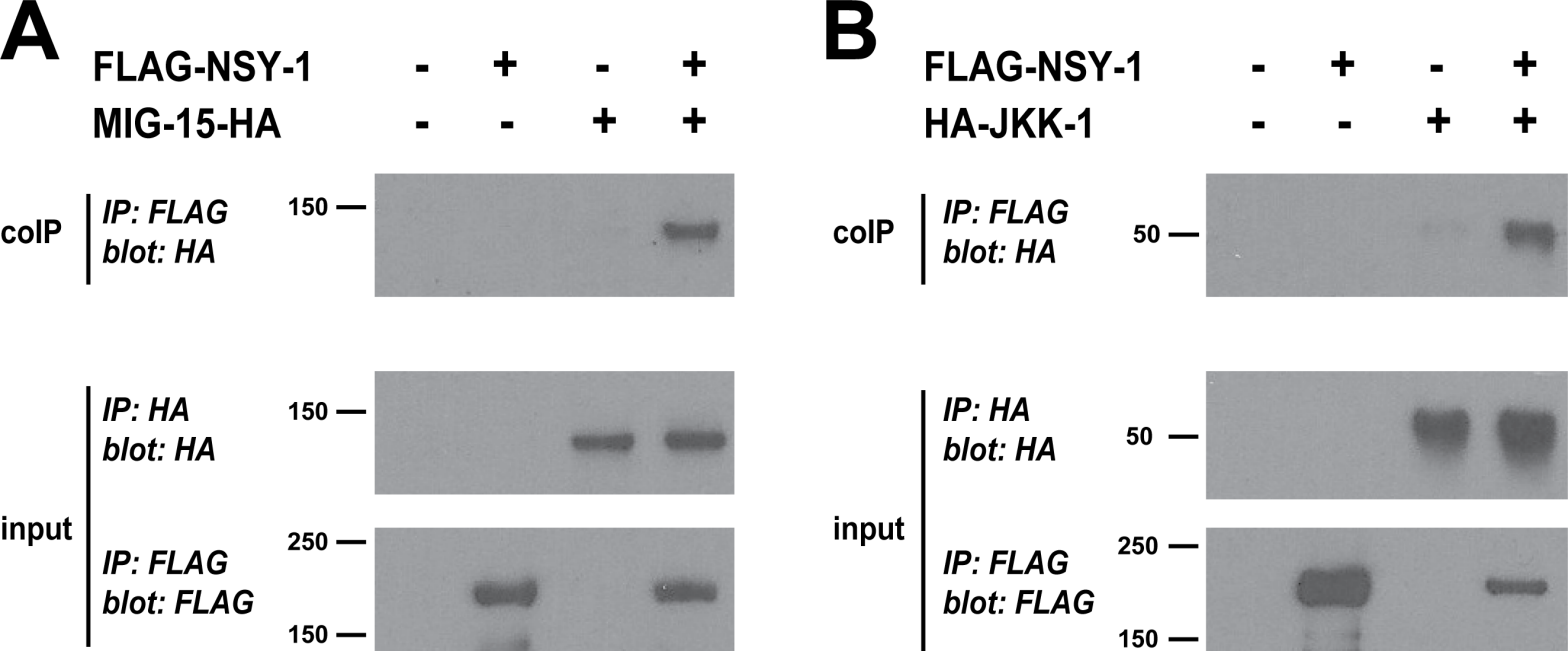
NSY-1 binds to MIG-15 and JKK-1. (**A**) coIP from HEK 293 showing MIG-15-HA coprecipitates with FLAG-NSY-1. (**B**) coIP from HEK 293 cells showing HA-JKK-1 coprecipitates with FLAG-NSY-1. Shown are representatives of at least 3 independent experiments.

Previous *in vitro* experiments showed MAP3Ks often bind to both MAP4Ks and MAP2Ks in the same pathway [60]. For example, MEKK1 (MAP3K1) binds to both the MAP4K NIK and the MAP2K MKK4 [46, 61], while NSY-1 binds to the MAP2K SEK-1 [62]. To test whether similar interactions occur between the NSY-1 MAP3K and the MIG-15 MAP4K or JKK-1 MAP2K, HEK 293 cells were transfected with FLAG or HA tagged kinases and binding was assessed by coimmunoprecipitation. When NSY-1 was immunoprecipitated from transfected cell lysates, we detected coprecipitating MIG-15 (Fig 7A) and JKK-1 (Fig 7B). NSY-1 binding to both MIG-15 and JKK-1 provides biochemical evidence that these kinases could function in a linear MAPK pathway.

### NSY-1 and JNK-1 localize to presynaptic terminals

**Fig 8:**
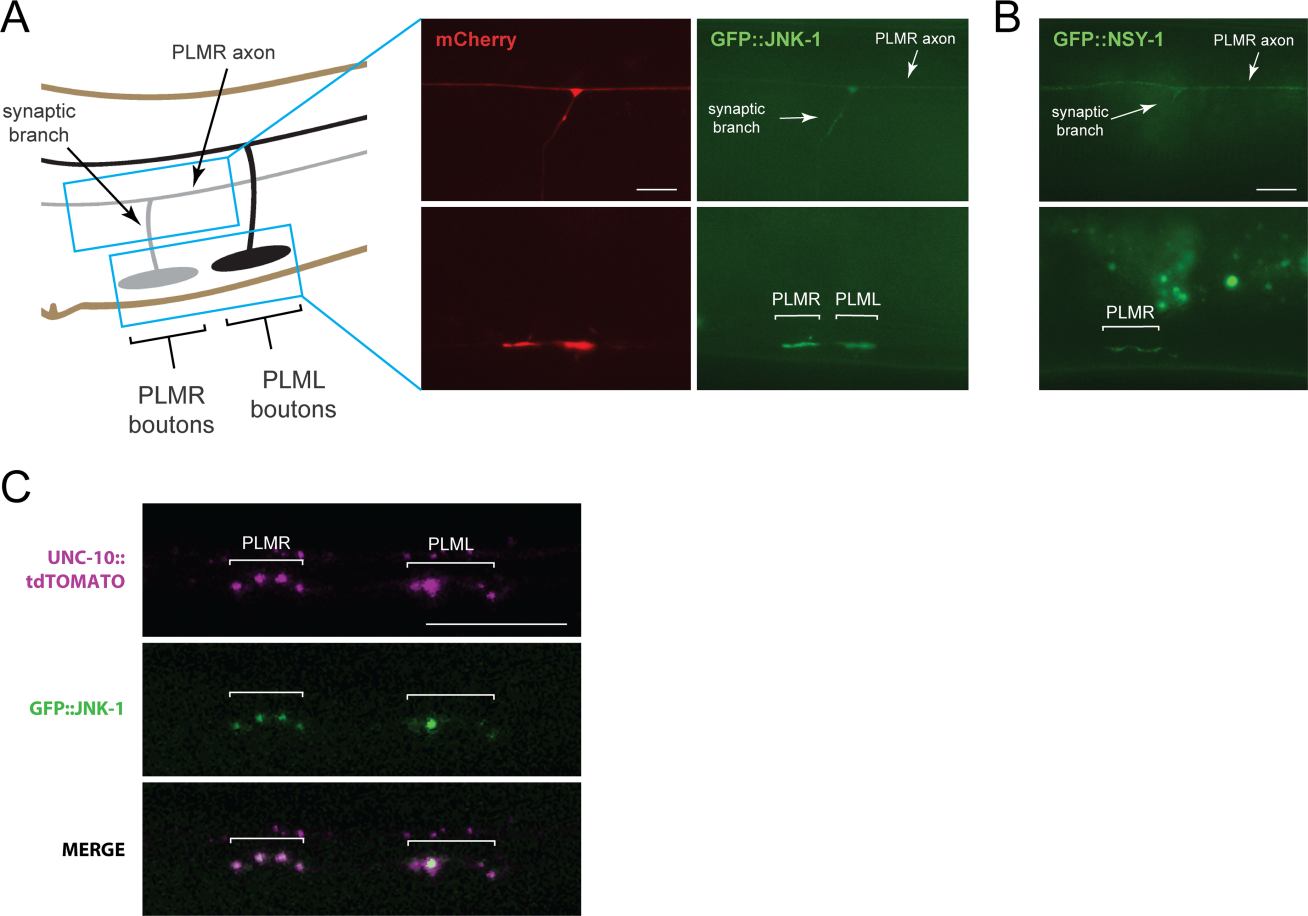
NSY-1 and JNK-1 localize to presynaptic terminals of PLM mechanosensory neurons. (**A**) Schematic depicts PLM axon, collateral synaptic branch, and presynaptic boutons. Blue box highlights regions imaged using epifluorescent microscopy. mCherry labels PLM neuron. GFP::JNK-1 diffusely localizes in axon (upper panel) and concentrates at presynaptic terminals (white brackets, lower panel). (**B**) GFP::NSY-1 at presynaptic terminals (white brackets). (**C**) Confocal projection shows GFP::JNK-1 colocalizes with UNC-10::tdTOMATO at presynaptic active zones of PLM neurons (white brackets). Scale bars 10 μm.

Because MIG-15, NSY-1, JKK-1 and JNK-1 affect synapse formation, it is plausible these kinases might localize to presynaptic terminals. To explore this, we generated transgenic extrachromosomal arrays that simultaneously expressed mCherry and GFP::JNK-1. As shown in Fig 8A, GFP::JNK-1 localized to the presynaptic boutons of PLM neurons. JNK-1 was also observed in the axon and synaptic branch. GFP::NSY-1 similarly localized to presynaptic boutons (Fig 8B). We tried to determine if MIG-15 and JKK-1 localize to presynaptic terminals, but these constructs were not readily detected in PLM neurons.

To further define presynaptic localization, we simultaneously expressed GFP::JNK-1 and the active zone marker UNC-10::tdTOMATO. Confocal microscopy showed colocalization between GFP::JNK-1 and UNC-10::tdTOMATO at presynaptic terminals (Fig 8C). This demonstrates that JNK-1 localizes to the presynaptic active zone of PLM mechanosensory neurons. Collectively, these results are consistent with NSY-1 and JNK-1 regulating glutamatergic synapse formation.

### *jkk-1* and *nsy-1* fail to suppress synapse formation defects in GABAergic motor neurons of *rpm-1* mutants

RPM-1 and its orthologs, Drosophila Highwire and mouse Phr1, regulate synaptogenesis at NMJs [16, 18, 19]. Loss of function in the DLK-1/PMK-3 or MLK-1/KGB-1 kinase pathway suppresses synapse formation defects in the GABAergic motor neurons of *rpm-1* mutants [29, 30]. Therefore, we tested whether NSY-1 and JKK-1 affect defects in GABAergic NMJs caused by *rpm-1* (lf).

**Fig 9:**
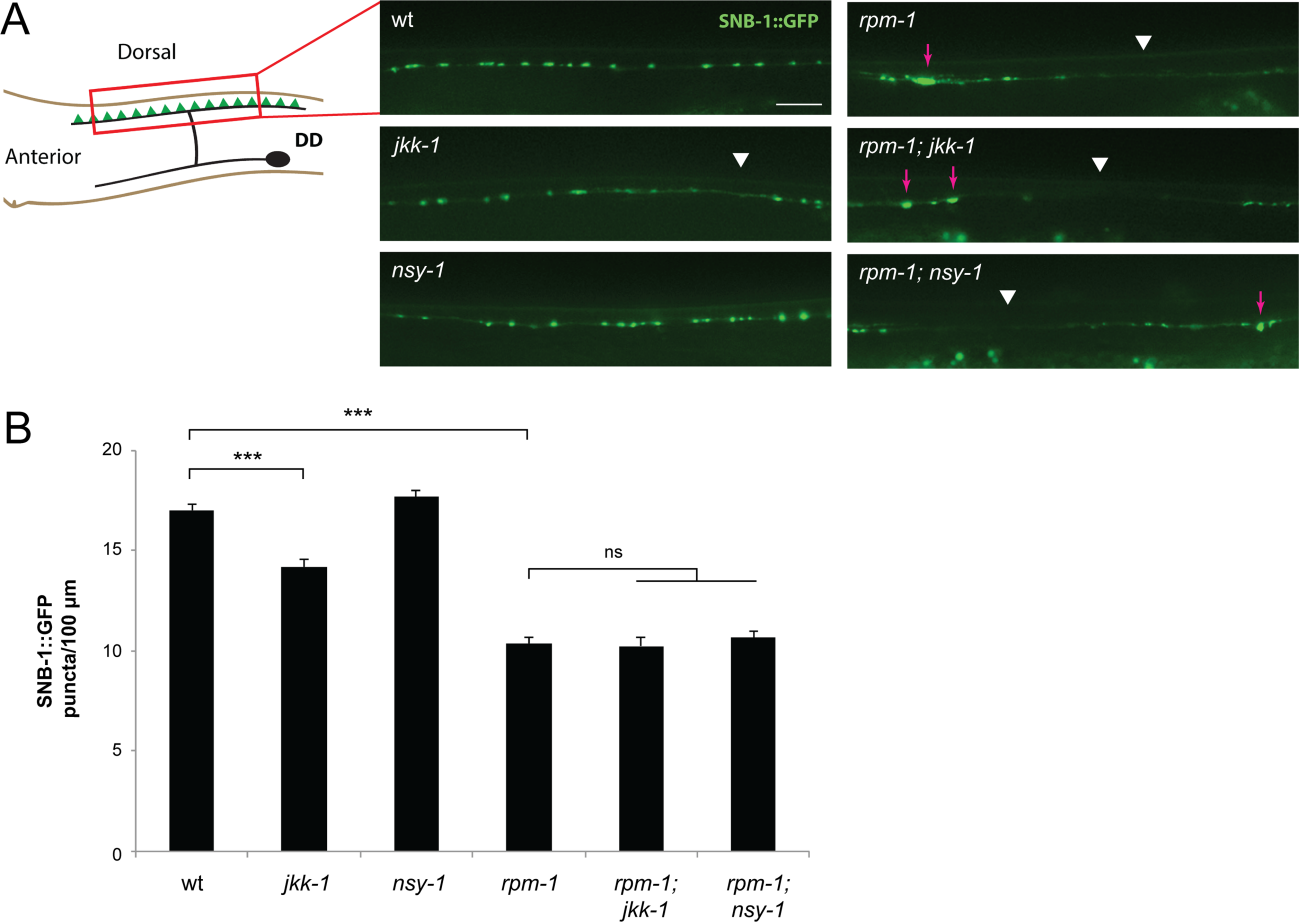
*jkk-1* and *nsy-1* do not suppress defects in NMJ synapse formation in motor neurons of *rpm-1* mutants. (**A**) Schematic shows GABAergic DD motor neuron with presynaptic terminals in green. Shown are images of SNB-1::GFP labeling GABAergic presynaptic terminals for indicated genotypes. Synapse formation defects, which present as aggregated SNB-1 puncta (arrows) and gaps in which puncta are absent (arrowheads), are highlighted in *rpm-1* single mutants as well as *rpm-1; jkk-1* and *rpm-1; nsy-1* double mutants. (**B**) Quantitation of SNB-1::GFP puncta in dorsal cord for indicated genotypes. Averages are shown for data collected from 3 or more independent experiments (27–36 worms/genotype). Error bars represent standard error of mean, and significance was determined using unpaired Student’s *t* test with Bonferroni correction. *** p< 0.001. Scale bar 10 μm.

The GABAergic DD motor neurons of *C*. *elegans* form synapses with dorsal body wall muscles (Fig 9A). To visualize GABAergic NMJs we used *juIs1*, a transgene that expresses the synaptic vesicle marker Synaptobrevin-1 fused to GFP (SNB-1::GFP). We observed regularly spaced presynaptic puncta in wild-type animals labeled with SNB-1 (Fig 9A). Consistent with previous studies, *rpm-1* mutants had synapse formation defects in which large sections of the dorsal cord lacked SNB-1 puncta, and SNB-1 puncta aggregated (Fig 9A). Interestingly, synapse formation defects were unchanged in *rpm-1; jkk-1* and *rpm-1; nsy-1* double mutants (Fig 9A). Quantitation of SNB-1 puncta confirmed synapse formation defects in GABAergic motor neurons of *rpm-1; jkk-1* or *rpm-1; nsy-1* double mutants were not suppressed compared to *rpm-1* single mutants (Fig 9B). We observed a small, significant defect in NMJ formation in *jkk-1* single mutants (Fig 9B). This suggests JKK-1 might regulate GABAergic synapse formation. Overall, these results demonstrate that NSY-1 and JKK-1 do not affect RPM-1 regulation of synapse formation in GABAergic motor neurons the way they do in PLM mechanosensory neurons. This indicates that loss of NSY-1 and JKK-1 specifically suppresses synapse formation defects at glutamatergic, neuron-neuron connections.

### *nsy-1*, *jkk-1* and *jnk-1* suppress tap habituation defects in *rpm-1* mutants

**Fig 10:**
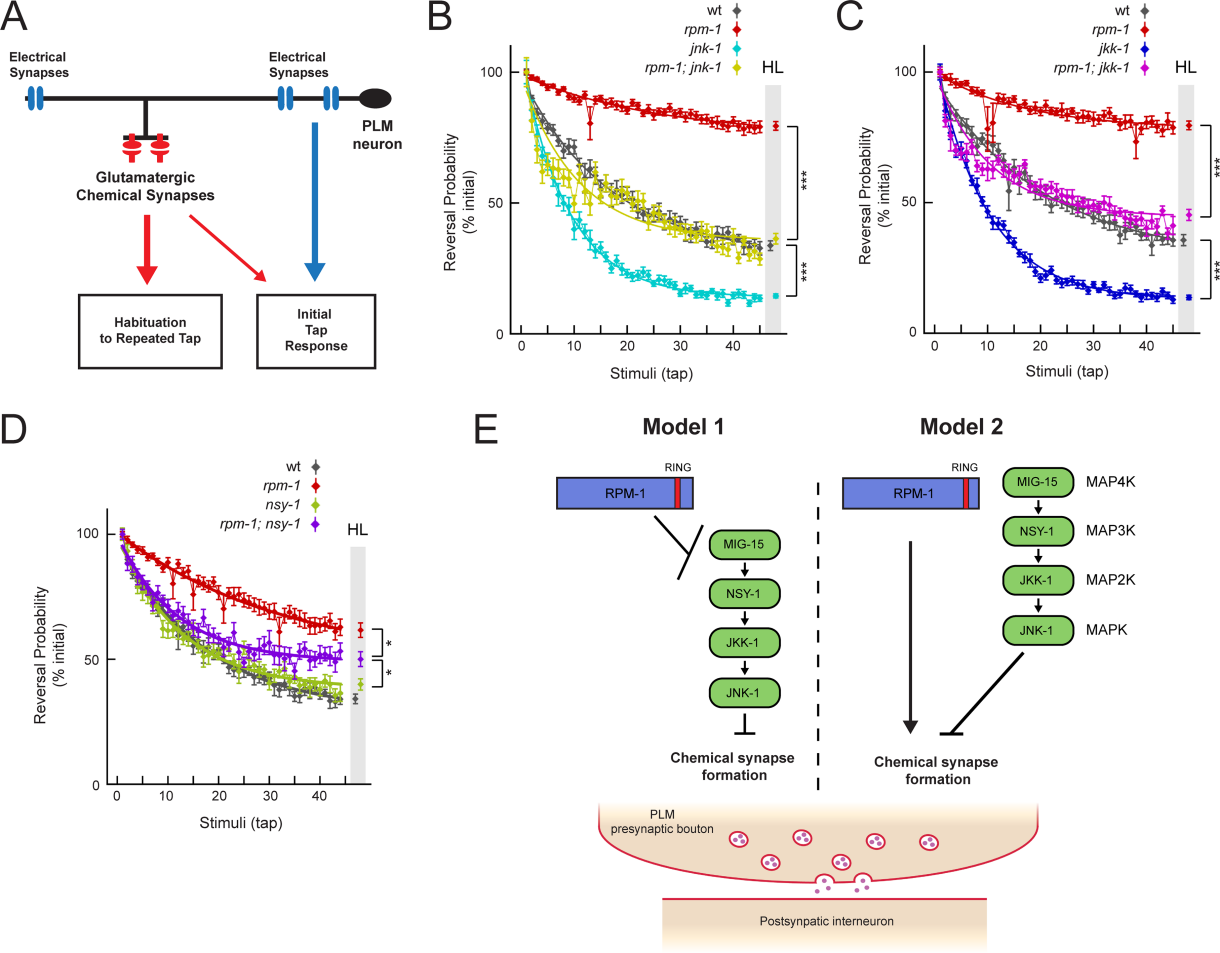
*jnk-1*, *jkk-1* and *nsy-1* suppress habituation defects in *rpm-1* mutants. (**A**) Schematic summarizing distinct anatomy and behavioral outputs of electrical (blue) and chemical (red) synapses in PLM mechanosensory neurons. (**B**) Tap habituation for indicated genotypes. *jnk-1* mutants have increased habituation and *rpm-1* mutants have decreased habituation. *rpm-1; jnk-1* double mutants (yellow) are suppressed and show intermediate habituation level compared to *rpm-1* and *jnk-1* single mutants. (**C**) Tap habituation for indicated genotypes. *rpm-1; jkk-1* double mutants (magenta) show suppression of tap habituation defects in *rpm-1* mutants, and have intermediate habituation level compared to *rpm-1* and *jkk-1* single mutants. (**D**) Tap habituation for indicated genotypes. *rpm-1; nsy-1* double mutants (purple) show suppression of tap habituation defects in *rpm-1* mutants, and have intermediate habituation level compared to *rpm-1* and *nsy-1* single mutants (**E**) Signaling models for how RPM-1 might relate to MIG-15/NSY-1/JKK-1/JNK-1 MAP kinase pathway in PLM neurons. Model 1, RPM-1 functions as an upstream inhibitor of MIG-15/JNK-1 pathway, most likely via RPM-1 ubiquitin ligase activity. Model 2, depicts MIG-15/JNK-1 pathway acting as a parallel opposing pathway to RPM-1. Outcomes from **B-D** support Model 2. Points and error bars represent mean ± SEM reversal probability (percent initial response) for 45 tap stimuli with 10 second inter-stimulus interval (n = 12 replicates of 50–100 animals from three independent experiments). Smooth lines are exponential best fit. Habituation level (HL, grey shaded area) is mean ± SEM value of exponential fit at final stimulus. Significance assessed by Student’s *t*-test. * *p* < 0.05 and *** *p* < 0.001.

The mechanosensory neurons mediate gentle touch responses, including the response to non-localized mechanical stimuli such as vibrations caused by tapping the plate on which worms grow. The mechanosensory neurons, including the PLM neurons, propagate sensory activity primarily via electrical synapses at sites along the primary axon (Fig 10A) [10, 63]. Glutamatergic chemical synapses are formed in an anatomically distinct location on the collateral synaptic branch, and contribute to a lesser extent to touch sensation (Fig 10A) [10, 11]. Glutamatergic transmission has been implicated in short-term learning, specifically habituation to repeated tap stimuli [13, 23].

Previous work showed *rpm-1* mutants respond normally to touch [5, 23], which is consistent with these animals having normal electrical synapses in their mechanosensory neurons [64] (Borgen and Grill, *in press*). In contrast, *rpm-1* mutants have dramatic defects in habitation to repeated tap, which result from loss of RPM-1 function in the mechanosensory neurons [23]. This suggests that habituation defects in *rpm-1* mutants are likely to result from defective chemical synapse formation. Thus, tap habituation is a reasonable behavioral readout to further test the genetic relationship between *rpm-1* and kinases in the MIG-15/JNK-1 pathway. Tap habituation also provided us with a whole-animal, physiological assay that is sensitive to impacts on chemical synapse formation in the mechanosensory neurons.

As shown in Fig 10B, wild-type animals displayed habituation to repeated tap, in which responses diminish with increasing tap stimuli over time. Consistent with prior findings [23], *rpm-1* mutants were defective in habituation, and maintained a high probability of response over repeated tap stimuli (Fig 10B). Tap habituation defects were suppressed in double mutants of *rpm-1* with *jnk-1*, *jkk-1* or *nsy-1* (Fig 10B, 10C and 10D). Defects in body morphology and locomotion prevented us from testing *mig-15* mutants. These behavioral findings show that loss of NSY-1, JKK-1 and JNK-1 suppress *rpm-1* in the context of tap habituation. This finding correlates nicely with our findings on chemical synapse formation in PLM neurons. It also provides further behavioral evidence that is consistent with NSY-1, JKK-1 and JNK-1 functioning in the same pathway.

Our observation that impairing kinases in the MIG-15/JNK-1 pathway suppresses *rpm-1* (lf) could be explained by two different signaling models. First, RPM-1 might function as a ubiquitin ligase to inhibit the MIG-15/JNK-1 pathway (Fig 10E, Model 1), similar to what occurs with the DLK-1/PMK-3 and MLK-1/KGB-1 pathways [29, 30]. Alternatively, RPM-1 could function in a parallel opposing pathway to MIG-15/JNK-1 (Fig 10E, Model 2). Our unexpected observation that *jnk-1* and *jkk-1* single mutants have more rapid habituation, the opposing phenotype to *rpm-1*, provided us with an ideal phenotypic relationship to test genetic epistasis (Fig 10B and 10C). *rpm-1; jnk-1* and *rpm-1; jkk-1* double mutants had an intermediate habituation phenotype similar to wild-type animals (Fig 10B and 10C). This suggests it is likely the MIG-15/JNK-1 pathway functions in a parallel opposing pathway to RPM-1 (Fig 10E, Model 2). It is not clear to us why *nsy-1* mutants did not have increased habituation. One possibility is another, unknown MAP3K functions redundantly with NSY-1 to regulate JKK-1. Nonetheless, we also observed an intermediate habituation phenotype in *rpm-1; nsy-1* double mutants (Fig 10D). Collectively, these habituation results with JNK-1, JKK-1 and NSY-1 are consistent with the MIG-15/JNK-1 pathway functioning as a parallel opposing pathway to RPM-1 (Fig 10E, Model 2).

## Discussion

Previous studies have shown the *C*. *elegans* PHR protein RPM-1 functions through multiple mechanisms to regulate synapse formation and axon termination [15]. One mechanism is RPM-1 acting as a ubiquitin ligase to inhibit p38 and JNK signaling pathways. It remains unclear how broad a regulator of p38 and JNK signaling RPM-1 is. Furthermore, it is unknown if there are pathways that interact with RPM-1 to specifically affect synapse formation.

Here we identify several kinases, that are likely to form a MAP kinase pathway, that specifically suppresses synapse formation defects caused by *rpm-1* (lf) in the mechanosensory neurons. This pathway is composed of the MIG-15 MAP4K, the NSY-1 MAP3K, the JKK-1 MAP2K and the JNK isoform JNK-1 (Fig 10E). Mutations in kinases of the MIG-15/JNK-1 pathway suppress synapse formation defects in the mechanosensory neurons of *rpm-1* mutants, and suppress accompanying deficits in habituation. Furthermore, behavioral analysis is consistent with the MIG-15/JNK-1 pathway most likely functioning as a parallel opposing pathway to RPM-1. Thus, our results suggest the MIG-15/JNK-1 pathway needs to be opposed by RPM-1 signaling for proper glutamatergic synapse formation and short-term learning (Fig 10E, Model 2). Our results also address an important missing functional link between the MIG-15 MAP4K and JNK in the nervous system.

### MIG-15/JNK-1 pathway and synapse formation

Biochemical studies and genetics on fly embryogenesis showed the orthologs of MIG-15 (Msn and NIK) activate JNK signaling [46, 47]. In worms and flies, MIG-15 and Msn regulate axon guidance by functioning through the cytoskeletal regulators, ERM-1 and Bifocal, rather than acting on JNK signaling [48, 49]. At present, it is unknown if MIG-15, Msn or NIK function through JNK signaling in the nervous system.

We now provide evidence that MIG-15 is likely to function in a JNK pathway to regulate synapse formation in mechanosensory neurons. If this is the case, in the developing worm nervous system MIG-15 regulates axon guidance through the actin regulator ERM-1, and regulates synapse formation through a kinase pathway that includes the JNK isoform JNK-1. Our results show *mig-15* mutants have mild defects in synapse formation, and suppress the strong synapse formation defects caused by *rpm-1* (lf) (Figs 2 and 3). This suggests a balance of MIG-15 signaling is important for proper glutamatergic synapse formation. The importance of our finding is accentuated by recent work showing NIK potentially mediates synapse loss during neurodegeneration in ALS [65].

Numerous observations collectively support the conclusion that *mig-15*, *nsy-1*, *jkk-1* and *jnk-1* function in the same pathway. 1) Suppression of synapse formation defects caused by *rpm-1* is not increased when multiple kinases are eliminated compared to elimination of a single kinase (Figs 1 and 2). 2) The MIG-15/JNK-1 pathway has a specific suppressor phenotype: These kinases suppress synapse formation defects, but not axon termination defects, in the mechanosensory neurons of *rpm-1* mutants (Figs 1 and 2). 3) Kinases in the MIG-15/JNK-1 pathway function cell autonomously in the mechanosensory neurons (Fig 5). 4) Transgenic overexpression of MIG-15, NSY-1 or JKK-1 causes synapse formation defects at a similar frequency (Fig 6). 5) *nsy-1*, *jkk-1* and *jnk-1* suppress defects in tap habituation in *rpm-1* mutants (Fig 10). 6) Finally, our biochemical results show that NSY-1 binds to MIG-15 and JKK-1 (Fig 7). Our findings on the MIG-15/JNK-1 pathway and the order we have assigned to kinases in the pathway agrees with prior *in vitro* biochemistry, genetic results on fly embryogenesis, and is consistent with how MAPK cascades generally operate [45]. However, our results do not definitively rule out the alternative possibility that theses kinases could function in multiple, parallel MAPK pathways. This is important to note, as prior work has shown that NSY-1 can stimulate activation of p38 MAPK [55].

Prior work showed JKK-1 and JNK-1 function in the same pathway to regulate locomotion, synaptic vesicle trafficking and ARL-8 regulation of synaptic position [51, 52]. Our results now identify a new function for JKK-1 and JNK-1 in glutamatergic synapse formation, and suggest MIG-15 and NSY-1 are likely to be further kinases in this pathway. It is unlikely impaired synaptic vesicle trafficking in *jnk-1* and *jkk-1* mutants would explain our findings, as we observe the opposite phenotype, an improvement in synapse formation defects in *rpm-1; jkk-1* and *rpm-1; jnk-1* double mutants. However, if synaptic vesicle trafficking to glutamatergic presynaptic terminals is increased in *jnk-1* and *jkk-1* mutants, which remains unknown, it might explain our results. Whether MIG-15 and NSY-1 are involved in ARL-8 regulation of synaptic position like JKK-1 and JNK-1 remains an open question.

### MIG-15/JNK-1 pathway molecularly distinguishes axon termination from synapse formation

We have found that the MIG-15/JNK-1 pathway specifically suppresses synapse formation defects in the mechanosensory neurons of *rpm-1* mutants. This differs from prior work showing the DLK-1/PMK-3 and MLK-1/KGB-1 pathways suppress both axon termination and synapse formation defects in mechanosensory neurons, as well as synapse formation defects in motor neurons of *rpm-1* mutants [29, 30]. Because mechanosensory neurons form glutamatergic, neuron-neuron synapses that are reminiscent of mammalian central synapses, it is reasonable to speculate that the MIG-15/JNK-1 pathway might regulate central synapse formation in other organisms.

Recent studies suggested axon termination defects are a remodeling phenotype that occurs due to retraction of the PLM synaptic branch [59, 66]. This is one possible explanation for why mutants, such as *rpm-1*, affect both axon termination and synapse formation. However, if this were true, we would expect genetic changes to affect both axon termination and synapse formation in all cases. Our discovery of the MIG-15/JNK-1 pathway demonstrates distinct signals exist within the mechanosensory neurons that differentially influence chemical synapse formation compared to axon termination.

### MIG-15/JNK-1 pathway likely functions as a parallel opposing pathway to RPM-1

Accompanying differing effects of the MIG-15/JNK-1 pathway and the DLK-1/PMK-3 pathway on synapse development are differing results on habituation to tap, a form of short-term learning, that is dependent upon chemical synapse function in the mechanosensory neurons. Prior work showed *rpm-1* mutants have dramatic defects in habituation to repeated tap stimulus, which is suppressed by *dlk-1* [23]. Defects in habituation stem from RPM-1 function in the mechanosensory neurons. We have found *nsy-1*, *jkk-1* and *jnk-1* also suppress habituation defects in *rpm-1* mutants. However, there were important differences between these kinases and DLK-1. First, *jkk-1* and *jnk-1* single mutants have increased habituation (Fig 10B and 10C), which was not observed in *dlk-1* mutants [23]. Second, our results show that double mutants of *rpm-1* with *jnk-1*, *jkk-1* or *nsy-1* are suppressed to an intermediate habituation phenotype with neither phenotype from single mutants dominating. The most likely explanation for these results is that the MIG-15/JNK-1 pathway functions as a parallel opposing pathway to RPM-1 (Fig 10E, Model 2). This differs from the DLK-1/PMK-3 pathway, which is inhibited by RPM-1 [29]. While we favor the parallel opposing pathway interpretation of our data, it is important to note our genetic results do not rule out that RPM-1 might ubiquitinate and inhibit a kinase in the MIG-15/JNK-1 pathway, in which case the MIG-15/JNK-1 pathway would function downstream of RPM-1 (Fig 10E, Model 1).

Our finding that the MIG-15/JNK-1 pathway is likely to function as a parallel opposing pathway to RPM-1 indicates that RPM-1 could be a relatively specific inhibitor of certain p38 and JNK pathways. This is consistent with prior work showing RPM-1 binds to the PP2C phosphatase PPM-2 to specifically inhibit DLK-1 [27]. Thus, our findings continue to support the concept that RPM-1 is a relatively sophisticated regulator of intracellular signaling, and not simply a general silencer of MAPK signaling [15, 27].

Our discoveries that the MIG-15/JNK-1 pathway inhibits glutamatergic synapse formation, and that impairing certain kinases in the MIG-15/JNK-1 pathway increases short-term learning have potentially important implications for neurodegenerative disease. This is particularly noteworthy, as a JNK inhibitor was shown to reduce synaptic dysfunction and improve cognitive outcomes in mouse models of Alzheimer’s disease [41–43], and recent work showed genetically impairing JNK activation improves outcomes in ALS and Alzheimer’s models [44]. The specificity of the MIG-15/JNK-1 pathway compared to the DLK-1/PMK-3 and MLK-1/KGB-1 pathways in worms suggests inhibitors of specific JNK isoforms could have differing efficacy in neurodegenerative disease models.

## Materials and methods

### Genetics & strains

The N2 isolate of *C*. *elegans* was used for all experiments and worms were maintained using standard procedures. The following mutant alleles were used in this study: *rpm-1* (*ju44*), *jnk-1* (*gk7*), *jkk-1* (*km2*), *nsy-1* (*ok593*), and *mig-15* (*mu342*). Genotyping was done by PCR and restriction digestion. *nsy-1 (ok593)* was maintained as a balanced strain with *mIn1*. *mig-15* mutants were maintained at 20°C and shifted to 23°C for experiments. The MosSCI insertion strain EG6699, *tTi5605* (Mos1 transposon on chromosome II); *unc-119 (ed3)*, was used to generate the integrated transgene *bggSi1* [P_mec-7_MIG-15) that was used for *mig-15* rescue experiments.

Other integrated transgenes used included: *muIs32* [P_mec-7_::GFP] II, *jsIs1114* [P_mec-7_GFP::RAB-3] II, *jsIs973* [P_mec-7_mRFP] III, *juIs1* [P_unc-25_SNB-1::GFP] IV and *bggIs28* [P_mec-7_::UNC-10::tdTOMATO] III.

### Cloning

cDNA encoding *jnk-1*, *jkk-1*, *nsy-1* or *mig-15* were cloned with iProof High-Fidelity DNA polymerase (BIO-RAD) PCR from an N2 cDNA pool using standard conditions. cDNAs were Taq polished and TOPO cloned into pCR8 Gateway entry vector (Invitrogen). cDNA entry vectors were validated by sequencing and recombined into the necessary Gateway destination vectors to create final plasmids for microinjection or transfection into HEK 293 cells.

For construction of P*mec-7*::UNC-10::tdTOMATO (pBG-GY757), tdTOMATO cDNA was initially subcloned by PCR, adding PpuMI and NheI sites to 5’ and 3’ ends, respectively. These enzyme sites were used to insert tdTOMATO into a P*mec-7* Gateway destination vector to create the C-terminal tagging vector pBG-GY747. *unc-10* genomic DNA was TOPO cloned using pJH430 (kind gift of Dr. Mei Zhen, University of Toronto) as a PCR template, and recombined into pBG-GY747.

To build a targeting vector for *mig-15* MosSCI insertion, the pCR8 *mig-15* Gateway entry vector (pBG-GY596) was recombined with a P*mec-7* destination vector (pBG-GY119). The *mec-7* promoter, *mig-15* cDNA, and *unc-54* 3’UTR was amplified by PCR as a single sequence using iProof DNA polymerase. SpeI sites were included in the primers and added to the 5’ and 3’ ends of this construct. PCR product was TOPO cloned into pCR8 to create pBG-GY616 and verified by sequencing. pBG-GY616 plasmid was cut with SpeI and subcloned into the pCFJ350 MosSCI targeting vector.

### Transgenics

Transgenic strains were derived using standard procedures. For mechanosensory neuron rescue experiments, plasmid DNA mixtures were injected in double mutants of interest with *muIs32* to derive transgenic extrachromosomal arrays that used the *mec-7* promoter to drive expression from *jnk-1*, *jkk-1* or *nsy-1* cDNA. *mig-15* rescue was done using a MosSCI single copy integrated transgene, *bggSi1*, that was crossed onto *rpm-1* (*ju44*); *mig-15* (*mu342*) double mutants. MosSCI was necessary due to technical limitations of injecting into *mig-15* mutants, which have small body and brood size. MosSCI insertion was carried out with *peel-1* negative selection [67].

For overexpression experiments, kinase cDNAs were expressed using the pan-neuronal *rgef-1* promoter and extrachromasomal arrays were derived using wild-type animals. PCR amplification was performed using the Roche Expand Long Template PCR system.

For localization experiments, transgenic extrachromosomal arrays were generated in wild-type animals by injecting plasmids that used the *mec-3* or *mec-7* promoter to express *nsy-1* or *jnk-1* cDNA with N-terminal GFP fusions. For colocalization, GFP::JNK-1 was injected into *bggIs28*. The *bggIs28* transgene was generated by TMP/UV genomic integration of a transgenic array containing P*mec-7*::UNC-10::tdTOMATO (injected at 25 ng/μL). We generated N and C-terminal GFP and tdTOMATO fusions with MIG-15 and JKK-1, but these constructs were not readily detected in PLM neurons across different injection concentrations using either the *mec-3* or *mec-7* promoters.

All injection mixes used to make extrachromasomal arrays included the co-injection marker P*myo*-2::mCherry at 1 ng/μL, and were made up to total DNA concentration of 100 ng/μL using pBluescript. S1 Table contains information for DNA concentrations for all individual injections. DNA was microinjected with using Zeiss AxioObserver.A1 microscope, Shutter Instrument XenoWorks Digital Microinjector and NARISHIGE MO-202U micromanipulator and standard injection procedures.

### Analysis of axon and synapse development

PLM axon termination and synaptic branch morphology was analyzed and quantified using the transgene *muIs32*. L4 parent worms were grown at 23°C and F1 progeny were picked to separate plates as L4 animals and maintained at 23°C. Animals were scored or imaged 16–24 hours later as young adults. This procedure was used to ensure consistent developmental staging across genotypes. Animals were anesthetized in 5 μM levamisole or 1% (v/v) 1-phenoxy-2-propanol in M9 buffer on a 2% agarose pad, and mounted and visualized with a Leica DM5000 B (CTR5000) epifluorescence microscope using 40x or 63x oil immersion objectives. Images were acquired with a CCD Leica DFC345 FX camera.

The synaptic branch was scored as a proxy for synapse formation in the PLM neurons. Defects were scored by following PLM synaptic branches down to the ventral nerve cord (VNC). Branches with terminal GFP varicosities, representing presynaptic boutons, were scored as wildtype. The absence of terminal varicosities and incomplete branches, or total absence of branches were scored as mutant. A subset of *mig-15* mutants had multiple collateral PLM branches, a phenotype previously documented in the DD motor neurons for *mig-15* mutants [48]. If at least one complete branch reached the VNC to form a varicosity the neuron was not counted as mutant for synaptic branch. The position of the vulva was also used to assess which branch was the normal, wild-type synaptic branch in these animals. A small proportion of *mig-15* mutants displayed severe migration and guidance problems; the PLM neurons from these neurons were not included in our analysis.

To score PLM neurons simultaneously expressing P*mec-7*::GFP::RAB-3 (*jsIs1114*) and P*mec-7*::mRFP (*jsIs973*), the same criteria for the synaptic branch was used as above, but the presence of GFP::RAB-3 puncta was scored simultaneously. Whenever GFP::RAB-3 puncta were observed at the presynaptic terminal, the PLM synaptic branch and presynaptic bouton morphology were also normal.

Axon termination was scored as mutant if the axon overextended beyond the normal termination point (prior to the ALM cell body) and curved towards the VNC. These defects were referred to as “hook” defects.

To evaluate synapse formation in the GABAergic motor neurons, we used the transgene P*unc-25*::SNB-1::GFP (*juIs1*). Analysis of SNB-1::GFP puncta was done on young adult worms grown at 25°C. Worms were prepared for analysis as described for the PLM neurons, and anesthetized with 1% (v/v) 1-phoxy-2-propanol. Images of the dorsal cord were collected using a 40x oil immersion objective. Stretches of dorsal cord were measured using Leica Application Suite AF software, and the number of SNB-1::GFP puncta were manually counted.

### Confocal microscopy

L4 worms were picked to a separate plate and young adults were imaged 18–24 hours later. Slides were prepared with the same procedure described for PLM analysis with cytosolic GFP using 5 μM levamisole. Coverslips were sealed with BIOTIUM CoverGrip Coverslip Sealant. Confocal images were collected with Leica SP8 confocal microscope under 25x or 40x objectives. The following acquisition conditions were used: Bidirectional, 1.00 AU pinhole, 2.5–3.0x scan zoom factor, 400-600Hz, HyD detectors, 150–200 gain, 512x512 format, between lines sequential acquisition. Analysis conditions were kept identical across genotypes. Leica Application Suite (LAS) software was used to define Z-stacks that were collected at 1.0 to 1.5μm intervals. Maximum intensity projections of Z-stacks were made using LAS software.

### Presynaptic morphology analysis

Exported TIFF files of confocal stacks were analyzed in FIJI (ImageJ). Regions of interest (ROI) were defined manually for GFP::RAB-3 and UNC-10::tdTOMATO puncta within PLM presynaptic boutons. Mean area and number of puncta were calculated based on ROIs, and data was pooled from three separate experiments for each genotype.

### Colchicine treatment

Colchicine (0.25 mM from a 0.5 M stock solution dissolved in DMSO) was added while pouring NGM plates. Control NGM plates were made by adding an equal volume of DMSO. OP50 bacteria lawns were seeded onto colchicine or DMSO plates and used within 10 days of pouring. P0 young adult worms were placed on colchicine or DMSO plates, and F1 young adults were analyzed.

### Biochemistry

6-cm dishes of confluent HEK 293-T cells were transfected with 10 μL of Lipofectamine using the designated amount of plasmid: pBG-GY711 (FLAG-NSY-1, 2 μg), pBG-GY738 (MIG-15-HA, 4 μg) and pBG-GY784 (HA-JKK-1, 4 μg). pBluescript was added to reach 8 μg of total DNA transfected per plate. 22–26 hrs after transfection, cells were lysed with 0.1% NP-40 lysis buffer (50 mM Tris, pH 7.5, 150 mM NaCl, 10% glycerol, 1 mM DTT, and 1x Pierce HALT protease inhibitor cocktail). 0.25 to 0.5 mg of total protein from transfected cells was used for coIPs. Lysates were incubated with primary antibody for 30 min and precipitated for 4 h with 10 μL protein G agarose (Roche Applied Science) at 4°C. Precipitates were boiled in Laemmli sample buffer (Bio-Rad) with ß-mercaptoethanol (Sigma) and run on a 4–12% Bis-Tris gel (Invitrogen). Gels were transferred to PVDF membranes using Tris acetate transfer buffer and immunoblotted. Blots were visualized with HRP conjugated secondary antibodies, ECL (1:5 dilution of Supersignal West Femto (Thermo Scientific) in TBS) and x-ray film.

FLAG tagged proteins were immunoprecipitated with a mouse monoclonal anti-FLAG antibody (M2, Sigma), and immunoblotted with rabbit polyclonal anti-FLAG antibodies (Cell Signaling). HA tagged proteins were immunoprecipitated with rabbit polyclonal anti-HA antibodies (Invitrogen) and immunoblotted with a rabbit monoclonal anti-HA antibody (C29F4, Cell Signaling). For anti-HA blots, anti-rabbit light chain specific secondary antibodies were used.

### Habituation

Tap habituation experiments were performed as described previously with minor modifications [23]. Behavioral recordings were collected using a modified Multi-Worm Tracker. Age-synchronized animals (~50–100) were cultivated from egg until gravid adult (~3 days) at 23°C, and assayed on 5 cm NGM plates with 50 μl of OP50 *E*. *coli*. Behavior of animals was recorded for 550 seconds, and animals were mechanically stimulated by tapping the side of the plate with an automated linear solenoid after the first 100 seconds. Plates were tapped 45 times with a 10 second inter-stimulus interval. Response was measured as reversal probability, which was estimated by the proportion of worms that reversed within 2 seconds of each tap stimulus. Because reversal probabilities to the initial response differed in some genotypes (mean ± SEM, Fig 10B: wildtype = 0.90 ± 0.01, *rpm-1* = 0.98 ± 0.01, *jnk-1* = 0.78 ± 0.03, *rpm-1; jnk-1* = 0.53 ± 0.03; Fig 10C: wildtype = 0.89 ± 0.02, *rpm-1* = 0.96 ± 0.01, *jkk-1* = 0.81 ± 0.02, *rpm-1; jkk-1* = 0.75 ± 0.01; and Fig 10D: wildtype = 0.90 ± 0.01, *rpm-1* = 0.93 ± 0.01, *nsy-1* = 0.80 ± 0.02, *rpm-1; nsy-1* = 0.82 ± 0.02), responses were standardized as the percent mean of initial response. For each plate, exponential curves were fit to the responses across stimuli, and habituation level was measured as the value of the fit at the final stimulus. All strains used contained the *muIs32* transgene.

### Statistical analysis

#### PLM synapse formation and axon termination analysis

For analysis with *muIs32*, statistical comparisons were done with an unpaired, 2-tailed Student’s *t*-test and Bonferroni correction. Significance was defined as p<0.05. Bar graphs represent averages from 4–10 counts (25–35 neurons/count) for each genotype from three or more independent experiments. For analysis of the synaptic branch and RAB-3::GFP accumulation, bar graphs represent averages from 6–8 counts (25–35 neurons/count) for each genotype from three or more independent experiments. For transgenic experiments, bar graphs represent averages for data from 4–6 independent transgenic lines for each genotype. Error bars represent the standard error of the mean.

#### GABAergic synapse formation

For *juIs1* analysis, statistical comparisons were done with an unpaired, 2-tailed Student’s *t*-test and Bonferroni correction. Significance was defined as p<0.05. Bar graphs represent averages from 27–36 total animals collected from 3 or more independent experiments for each genotype. Error bars represent the standard error of the mean.

#### Habituation

One plate of animals was considered n = 1, and four plates were tested in three independent experiments for each genotype (n = 12). Graphs show the mean ± SEM of responses to all stimuli, an exponential fit of mean responses, and mean ± SEM for habituation level. Student’s *t*-tests were performed to assess statistical significance.

## Supporting information

S1 Fig Axon termination defects visualized with the transgene *jsIs973* are not suppressed in *rpm-1; jkk-1* and *rpm-1; mig-15* double mutants. Quantitation of axon termination defects (hook defects) for genotypes shown in Fig 3 using the transgene *jsIs973* (P*mec-7*::mRFP). Note there is no suppression of axon termination defects in *rpm-1; jkk-1* or *rpm-1; mig-15* double mutants. Shown are averages from 6–10 counts (25–30 neurons/count) of young adult animals for each genotype. Error bars represent standard error of mean, and significance was determined using unpaired Student’s *t* test with Bonferroni correction. ns = not significant.
(TIF)

S1 Table Transgenic DNA injection details. Worms of the indicated genotypes were injected with constructs at specified concentrations. Note that PCR prod. is linear DNA PCR product from Roche Expand Long Template PCR System (see Methods). Unless stated, all constructs contained *unc-54* 3’UTR sequence to aid neuronal expression. All transgenic constructs were injected with P*myo-2*::mCherry co-injection marker at 1 ng/μl (see Methods).
(DOCX)
