# Supplementary figures and images for: A MIG-15/JNK-1 MAP kinase cascade opposes RPM-1 signaling in synapse formation and learning
Source: PLoS Genet. 2017 Dec 11;13(12):e1007095. doi: 10.1371/journal.pgen.1007095 (PMC5754208; doi:10.1371/journal.pgen.1007095)

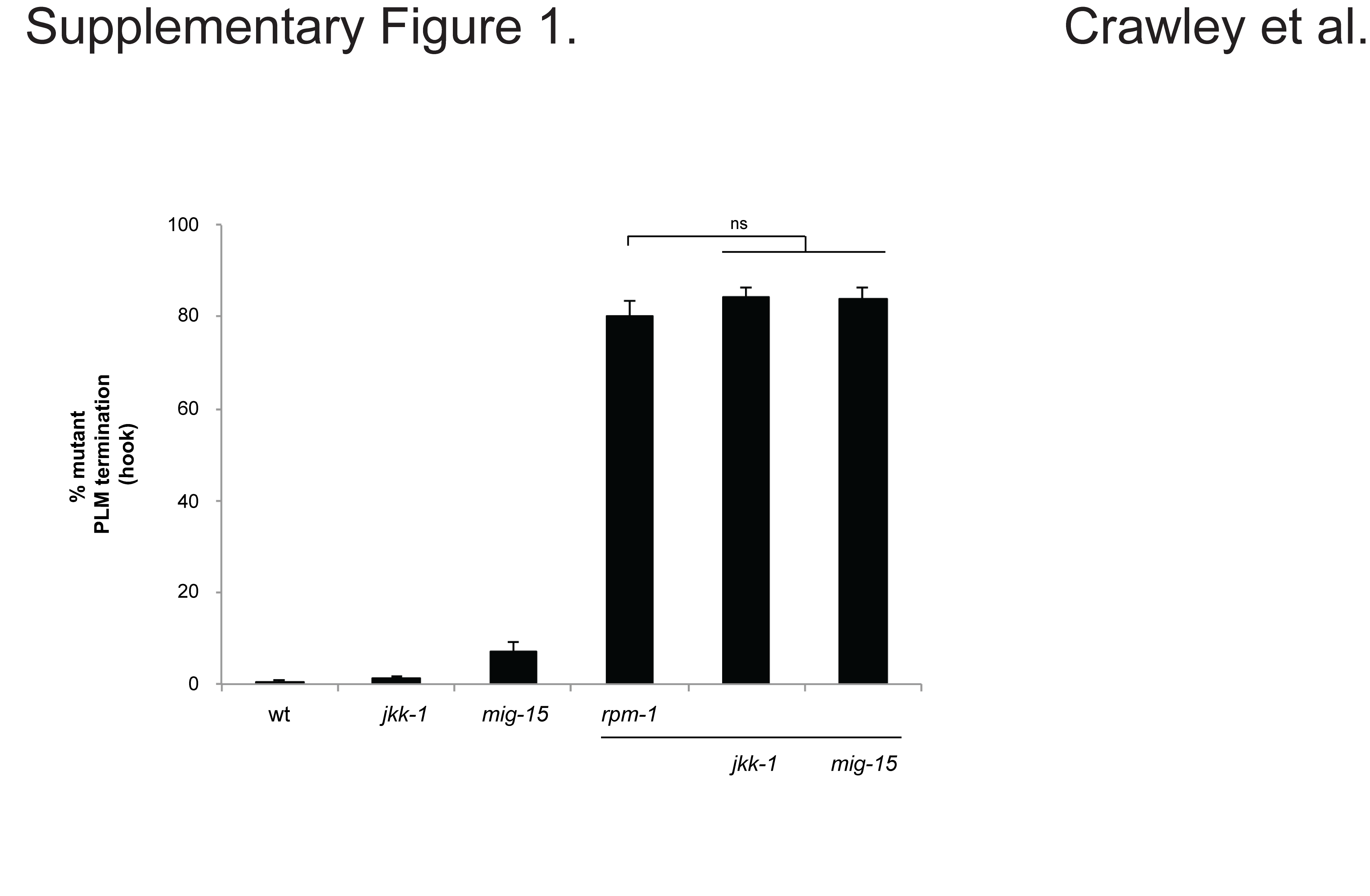

Supplement: S1 Fig — Quantitation of axon termination defects (hook defects) for genotypes shown in Fig 3 using the transgene jsIs973 (Pmec-7::mRFP). Note there is no suppression of axon termination defects in rpm-1; jkk-1 or rpm-1; mig-15 double mutants. Shown are averages from 6–10 counts (25–30 neurons/count) of young adult animals for each genotype. Error bars represent standard error of mean, and significance was determined using unpaired Student’s t test with Bonferroni correction. ns = not significant. (TIF) [file pgen.1007095.s001.tif]
