# Supplementary material for: A MIG-15/JNK-1 MAP kinase cascade opposes RPM-1 signaling in synapse formation and learning
Source: PLoS Genet. 2017 Dec 11;13(12):e1007095. doi: 10.1371/journal.pgen.1007095 (PMC5754208; doi:10.1371/journal.pgen.1007095)
Supplement: S1 Table — Worms of the indicated genotypes were injected with constructs at specified concentrations. Note that PCR prod. is linear DNA PCR product from Roche Expand Long Template PCR System (see Methods). Unless stated, all constructs contained unc-54 3’UTR sequence to aid neuronal expression. All transgenic constructs were injected with Pmyo-2::mCherry co-injection marker at 1 ng/μl (see Methods). (DOCX) [file pgen.1007095.s002.docx]

**Supplementary Table 1 – Transgenic DNA injection details**

| **Transgene**  **Name** | **Figure** | **Injection construct** | **Plasmid** | **Concentration (ng/µl)** | **Lines**  **Analyzed** | **Genotype background** |
| --- | --- | --- | --- | --- | --- | --- |
| - | 5A, B | P*mec-7*::JNK-1 | pBG-GY618 | 10 | 4 | *rpm-1; jnk-1; muIs32* |
| - | 5B | P*mec-7*::JKK-1 | pBG-GY632 | 10 | 6 | *rpm-1; jkk-1; muIs32* |
| - | 5B | P*mec-7*::NSY-1 | pBG-GY651 | 10 | 4 | *rpm-1; nsy-1; muIs32* |
| - | 5B, 8A | P*mec-7*::mCherry | pBG-GY258 | 10 (rescue)  60 (localization) | 5  - | *rpm-1; jnk-1; muIs32* |
| - | 6B | P*rgef-*1::JNK-1 | pBG-GY617 | 5  (PCR prod.) | 6 | wt |
| - | 6B | P*rgef-1*::JKK-1 | pBG-GY633 | 5  (PCR prod.) | 6 | wt |
| - | 6A, B | P*rgef-*1::NSY-1 | pBG-GY652 | 20  (PCR prod.) | 5 | wt |
| - | 6A, B | P*rgef-*1::MIG-15 | pBG-GY615 | 5  (PCR prod.) | 6 | wt |
| - | 6A, B | P*rgef-1*::mCherry | pBG-GY371 | 5 / 20  (PCR prod.) | 12 | wt |
| *bggEx128* | 8B | P*mec-3*::GFP::NSY-1 | pBG-GY708 | 25 | - | wt |
| *bggEx129* | 8A | P*mec-3*::GFP::JNK-1 | pBG-GY653 | 5 | - | wt |
| *bggEx130* | 8C | P*mec-7*::GFP::JNK-1 | pBG-GY793 | 20 | - | wt*;* *bggIs28* |
| MosSCI injection mix to make *bggSi1* | 5A, B | P*mec-*7::MIG-15 +  Cb-UNC*-119*(+)  (targeting vector)  P*eft-3*::Mos1  (trasnsposase)  P*hsp-16.41*::PEEL-1  P*rab-3*::mCherry  P*myo-2*::mCherry  P*myo-3*:mCherry | pBG-311  pCFJ601  pMA122  pGH8  pCFJ90  pCFJ104 | 22.5  50  10  10  2.5  5 | 1  (checked for full length  insertion) | EG6699  *ttTi5605 (II);*  *unc-119* (*ed3*) |
